# Changes in intestinal microbiota in postmenopausal oestrogen receptor-positive breast cancer patients treated with (neo)adjuvant chemotherapy

**DOI:** 10.1038/s41523-022-00455-5

**Published:** 2022-07-29

**Authors:** Romy Aarnoutse, Janine Ziemons, Lars E. Hillege, Judith de Vos-Geelen, Maaike de Boer, Saskia M. P. Bisschop, Birgit E. P. J. Vriens, Jeroen Vincent, Agnes J. van de Wouw, Giang N. Le, Koen Venema, Sander S. Rensen, John Penders, Marjolein L. Smidt

**Affiliations:** 1 GROW - School for Oncology and Developmental Biology, Maastricht University, Maastricht, the Netherlands; 2 Department of Surgery, Maastricht University Medical Centre, Maastricht, the Netherlands; 3 Department of Internal Medicine, Division of Medical Oncology, Maastricht University Medical Centre, Maastricht, the Netherlands; 4 Department of Medical Oncology, Catharina Hospital, Eindhoven, the Netherlands; 5 Department of Medical Oncology, Elkerliek Hospital, Helmond, the Netherlands; 6 Department of Medical Oncology, VieCuri Medical Centre, Venlo, the Netherlands; 7 Department of Medical Microbiology, Maastricht University Medical Centre, Maastricht, the Netherlands; 8 NUTRIM - School of Nutrition and Translational research In Metabolism, Maastricht University, Maastricht, the Netherlands; 9 Centre for Healthy Eating & Food Innovation, Maastricht University – campus Venlo, Venlo, the Netherlands; 10 Euregional Microbiome Center, Maastricht, the Netherlands

**Keywords:** Chemotherapy, Translational research

## Abstract

This clinical study explored the associations between the intestinal microbiota, chemotherapy toxicity, and treatment response in postmenopausal oestrogen receptor positive breast cancer patients.Oestrogen receptor positive postmenopausal breast cancer patients were prospectively enroled in a multicentre cohort study and treated with 4 cycles of (neo)adjuvant adriamycin, cyclophosphamide (AC) followed by 4 cycles of docetaxel (D). Patients collected a faecal sample and completed a questionnaire before treatment, during AC, during D, and after completing AC-D. Chemotherapy toxicity and tumour response were determined. Intestinal microbiota was analysed by amplicon sequencing of the 16 S rRNA V4 gene-region. In total, 44 patients, including 18 neoadjuvant patients, were included, and 153 faecal samples were collected before AC-D (*n* = 44), during AC (*n* = 43), during D (*n* = 29), and after AC-D treatment (*n* = 37), 28 participants provided all four samples. In the whole group, observed species richness reduced during treatment (*p* = 0.042). The abundance of Proteobacteria, unclassified Enterobacterales, *Lactobacillus*, *Ruminococcaceae NK4A214 group*, *Marvinbryantia*, *Christensenellaceae R7 group*, and *Ruminococcaceae UCG-005* changed significantly over time. Patients with any grade diarrhoea during docetaxel treatment had a significantly lower observed species richness compared to patients without diarrhoea. In the small group neoadjuvant treated patients, pathologic response was unrelated to baseline intestinal microbiota richness, diversity and composition. While the baseline microbiota was not predictive for pathologic response in a rather small group of neoadjuvant treated patients in our study, subsequent shifts in microbial richness, as well as the abundance of specific bacterial taxa, were observed during AC-D treatment in the whole group and the neoadjuvant group.

## Introduction

Breast cancer is the most common cancer in women worldwide^1^. Despite recent developments in systemic therapy, classical chemotherapeutic agents such as adriamycin, cyclophosphamide (AC) and docetaxel (D) remain the backbone of (neo)adjuvant chemotherapy regimes in postmenopausal oestrogen receptor-positive (ER+) breast cancer patients. Besides reducing tumour load in the neoadjuvant setting and improving disease-free and overall survival, AC-D treatment may induce toxicity, which impacts the quality of life and may require dose reductions. The most common non-haematological toxicities during adriamycin and cyclophosphamide treatment are oral mucositis, fatigue, alopecia, nausea and vomiting^2–4^. Docetaxel treatment shows a comparable toxicity profile with the addition of diarrhoea and peripheral sensory neuropathy^2–4^.

In order to reduce toxicity and optimise treatment outcomes, factors need to be identified that impact the individual response to and safety profile of AC-D. During the last decade, evidence of the interaction between systemic cancer therapies and the human intestinal microbiota has rapidly expanded^5,6^. The intestinal microbiota is an ecosystem that harbours trillions of intestinal microorganisms, consisting of bacteria, archaea, fungi, protozoa and viruses. It is well-established that crosstalk occurs between intestinal microbiota and the human host. This crosstalk is essential for the maintenance of immune function, homoeostasis and metabolism of dietary components and medication, including chemotherapeutic agents^7^. In the case of dysbiosis, intestinal microbiota can instigate carcinogenesis or affect systemic cancer therapy^8^.

Although interactions between AC-D and microbiota have not been studied in postmenopausal ER+ breast cancer patients, in vitro and mouse studies indicate that significant interactions occur between the intestinal microbiota and cyclophosphamide, adriamycin and docetaxel^9–16^. In mice, cyclophosphamide induces translocation of Gram-positive intestinal bacteria, including *Enterococcus hirae, Lactobacillus johnsonii* and *Lactobacillus murinus*, to mesenteric lymph nodes and the spleen. These bacteria, as well as *Barnesiella intestinihominis*, trigger an immune response and increase cyclophosphamide efficacy^9,11,15^. Furthermore, preclinical evidence has demonstrated an interaction between intestinal microbiota and adriamycin^12,16^. Rigby et al.^14^ concluded that the intestinal microbiota is necessary for adriamycin-induced intestinal damage and repair, but not for jejunal epithelial apoptosis. Limited pre-clinical evidence exists for interaction between docetaxel and intestinal microbiota^13^. Flórez et al.^10^ determined the susceptibility profiles of lactic acid bacteria and bifidobacteria to multiple chemotherapeutics and found that adriamycin perturbs the intestinal microbiota. Conversely, all tested members of the intestinal microbiota showed resistance to high doses of cyclophosphamide and docetaxel. However, these in vitro tests did not take into account the potential effect of in vivo transformation to more toxic compounds.

Despite the availability of the previously described pre-clinical evidence, no clinical studies with longitudinal microbiota sampling have yet explored the interaction between AC-D and the intestinal microbiota regarding chemotherapy toxicity and tumour response in postmenopausal ER+ breast cancer patients^17^. We hypothesise that the intestinal microbiota changes during AC-D treatment, and that the intestinal microbiota is associated with chemotherapy toxicity and tumour response in postmenopausal ER+ breast cancer patients.

## Results

### Baseline characteristics

**Fig. 1:**
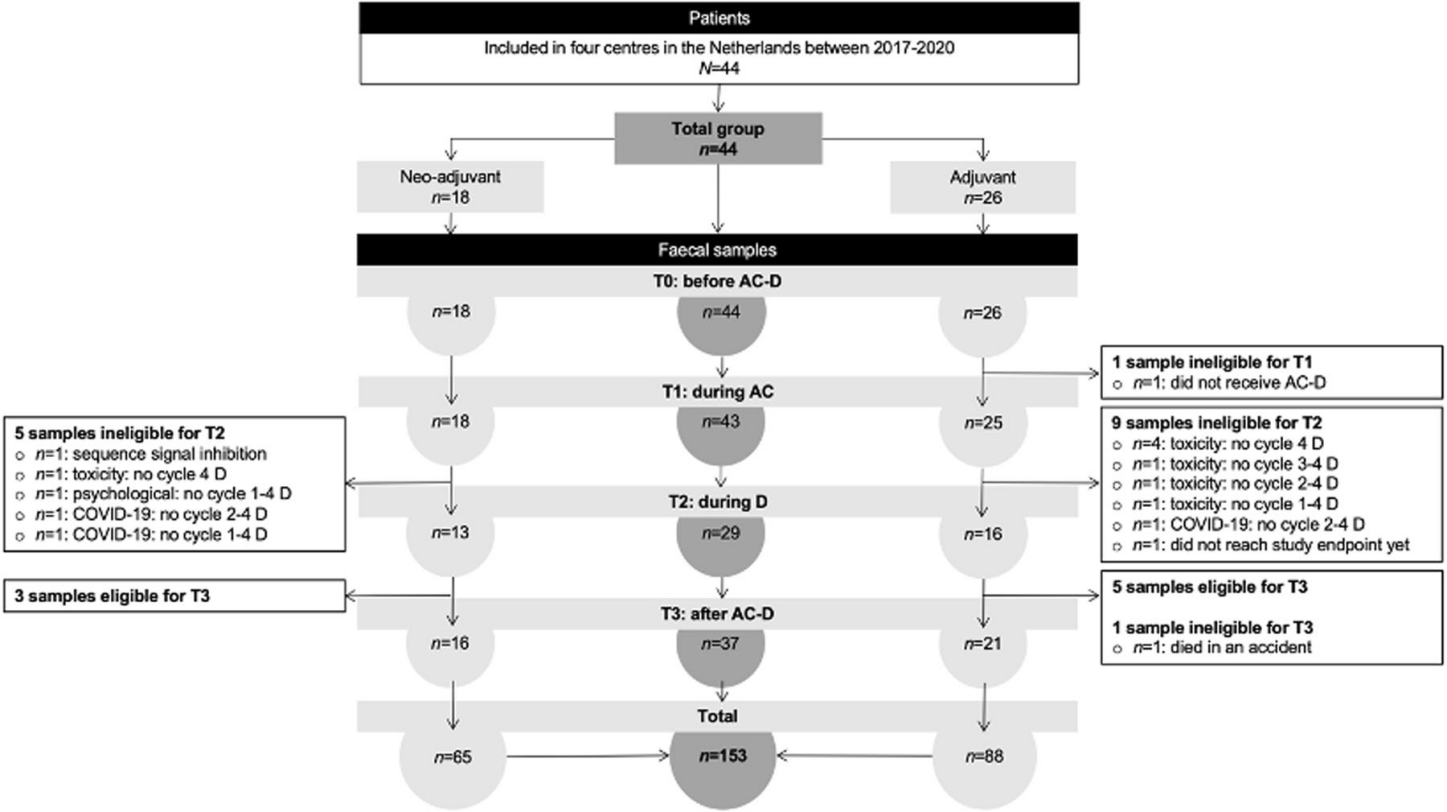
Flow chart. The flow chart presents the number of patients included and the number of faecal samples collected by those patients during the study period. Multiple patients who did not collect a faecal sample at T2 were able to collect a faecal sample at T3. In total, 44 patients collected 153 faecal samples at four-time points. 28 participants provided all four samples. The total group is presented in the middle. On the left and right sides, the total group is subdivided into neoadjuvant and adjuvant groups.

In total, 44 patients were included (Fig. 1). At baseline, mean age was 59 years. Mean BMI was 26 kg/m^2^. Nine per cent of the patients reported 5–10% weight loss during the previous 3–6 months before inclusion. Most patients were diagnosed with early-stage breast cancer.

In the year prior to inclusion, 27% of the patients used therapeutic antibiotics with a median use of seven days. None of the patients used therapeutic antibiotics within the three months prior to inclusion. The mean time between the last therapeutic antibiotic dose and baseline faecal sample collection was 31 weeks (range 15–52 weeks). Twelve (46%) adjuvant-treated patients received prophylactic cefazolin at the start of the operation. In addition, four (14%) of these twelve patients also received prophylactic amoxicillin/clavulanic acid for five days after the operation. The mean time between the operation and baseline faecal sample collection was 50 days. One patient used prebiotics in the year prior to inclusion. None of the patients used probiotics or nutritional supportive drinks in the year prior to inclusion. At baseline, neoadjuvant patients had higher clinical tumour stages and higher Karnofsky Performance Scores compared to adjuvant-treated patients (Table 1 and Supplementary Table 1).

**Table. 1:** Clinical characteristics of the total study population (*N* = 44) at baseline including the comparison between adjuvant- and neoadjuvant-treated patients.

Baseline characteristics	Total *n* = 44	Adjuvant *n* = 26	Neoadjuvant *n* = 18	*p* value
Age – Years				
Mean (SD)	59 (6)	59 (6)	58 (5)	0.478
BMI - kg/m^2^				
Median (IQR)	26 (5)	26 (4)	26 (7)	0.943
Weight loss past 3–6 months - in kg				
<5%	40 (91)	24 (92)	16 (89)	1.000
5–10%	4 (9)	2 (8)	2 (11)	
Clinical tumour stage - No (%)^1^				
Stage I	17 (40)	15 (58)	2 (12)	0.001
Stage II	23 (54)	11 (42)	12 (71)	
Stage III	3 (7)	0 (0)	3 (18)	
Tumour-type - No (%)				
Invasive carcinoma of no special type (NST)	33 (75)	17 (65)	16 (89)	0.089
Lobular	8 (18)	6 (23)	2 (11)	
Mucinous	2 (5)	2 (8)	0 (0)	
Unknown	1 (2)	1 (4)	0 (0)	
Therapeutic antibiotic use last year - No. (%)	12 (27)	8 (31)	4 (22)	0.733
Weeks between collection T0 faecal sample and last therapeutic antibiotic treatment				
Mean (SD)	31 (13)	29 (12)	33 (15)	0.713
Karnofsky Performance Score - No (%)*				
70–80	9 (21)	7 (27)	2 (11)	0.006
90–100	35 (79)	19 (73)	16 (89)	
MUST-score - No (%)				
Low risk	38 (86)	22 (85)	16 (89)	0.688
Medium risk	6 (14)	4 (15)	2 (11)	
High risk	0 (0)	0 (0)	0 (0)	
Oral contraception use past	34 (77)	19 (73)	15 (83)	0.489

^*^Percentages do not add up to 100% due to rounding.

### Clinical characteristics of patients during the course of AC-D treatment

During the course of AC-D treatment, patients had an increased risk of malnutrition (*p* < 0.001). The MUST-score improved in the period between T2 and T3 (*p* = 0.005). BMI remained stable over time (*p* = 0.338) (Supplementary Table 2).

Between T0-T1, 21% of the patients used antibiotics; 38% between T1 and T2 and 5% between T2 and T3. Most administered antibiotics included amoxicillin/clavulanic acid, nitrofurantoin and ciprofloxacin. None of the patients used prebiotics, probiotics, or nutritional supportive drinks during the course of AC-D treatment. In contrast to prophylactic antibiotic use prior to T0 faecal sample collection, antibiotic administration during AC-D treatment was not different between adjuvant and neoadjuvant treated patients (Supplementary Table 3).

Dose intensity was high, with a median of 94% of the chemotherapy dosage administered during AC-D treatment (Supplementary Table 4).

### Intestinal microbiota composition of the total study population

In total, 153 faecal samples were collected. Faecal samples were collected before AC-D (*n* = 44), during AC (*n* = 43), during D (*n* = 29) and after AC-D treatment (*n* = 37). 28 participants provided all four samples (Fig. 1).

**Fig. 2:**
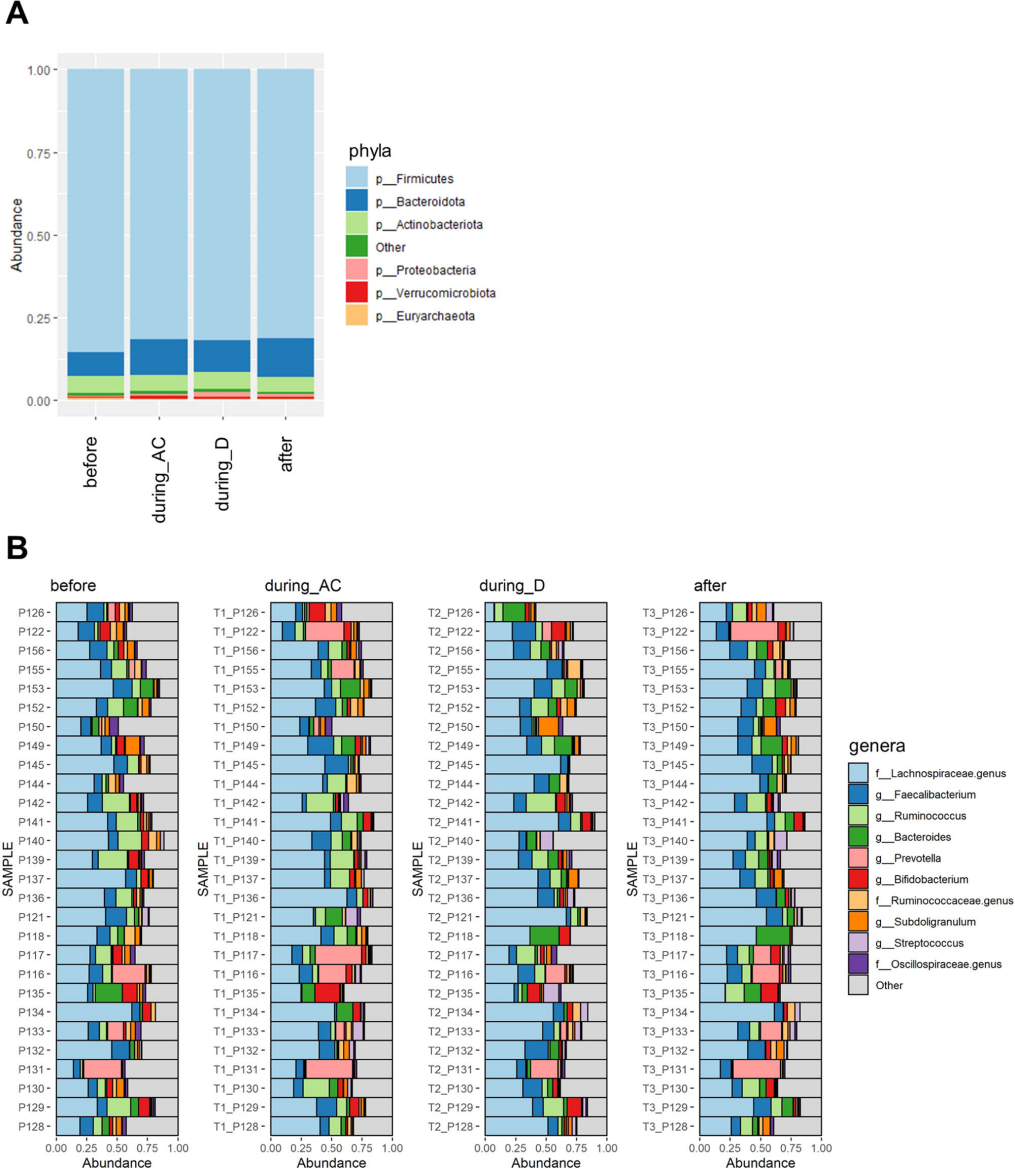
Relative abundances of microbiota before, during and after chemotherapy. **A** Relative abundances of different phyla before AC-D (*n* = 44), during AC (*n* = 43), during D (*n* = 29) and after AC-D treatment (*n* = 37). **B** Composition plot of individual samples (of participants who provided all four samples, *n* = 28) indicating changes in the relative abundance of most common genera over the course of AC-D treatment.

In the total study population, Firmicutes was the most abundant phylum, followed by Bacteroidetes and Actinobacteria (Fig. 2A). Figure 2B indicates changes in the relative abundance of the most common genera.

### Differences in microbiota richness, diversity and composition during the course of AC-D

**Fig. 3:**
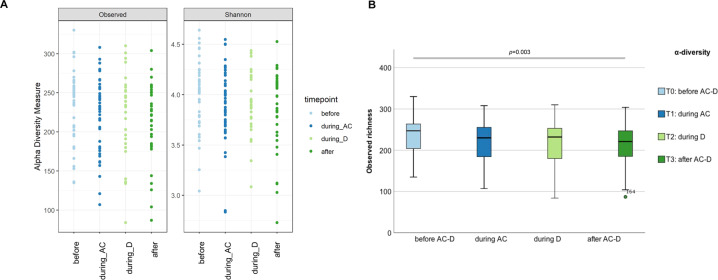
Microbiota diversity before, during and after chemotherapy. **A** Changes in α-diversity measures of the 28 participants who provided all four samples before AC-D, during AC, during D and after AC-D treatment, measured in terms of observed species richness (*p* = 0.042; *n* = 28) and Shannon index (*p* = 0.206; *n* = 28) (Supplementary Table 5). **B** Pairwise comparison (Wilcoxon signed-rank sum test with Bonferroni correction) of all samples before AC-D (*n* = 44), during AC (*n* = 43), during D (*n* = 29) and after AC-D treatment (*n* = 37) revealed significant differences in observed species richness between T0-T3 (*p* = 0.003; *n* = 37) (Supplementary Table 6). The boxplot in Fig. 3B shows the medians, IQR’s, minimum, maximum and an outlier.

Observed species richness reduced significantly during AC-D treatment (*p* = 0.042) (Fig. 3A and Supplementary Table 5). Pairwise comparison with Bonferroni correction of all samples revealed a significant decrease in observed species richness between T0 and T3 (*p* = 0.003; *n* = 37) (Fig. 3B and Supplementary Table 6).

Additional analyses were performed to assess the influence of exposure to therapeutic antibiotics before and during the course of AC-D treatment on α-diversity. Observed species richness and Shannon index before AC-D, during AC, during D and after D was not different between patients with or without therapeutic antibiotic use up to one year until three months prior to T0 (Supplementary Table 7A).

Antibiotic administration between T0 and T1 was negatively correlated with observed species richness (*p* = 0.002) and Shannon index (*p* = 0.003) at T1 (Supplementary Table 8). Cumulative therapeutic and prophylactic antibiotic use from the year prior to baseline faecal sample collection until the index sample, was not correlated with lower α-diversity at T1, T2 or T3 (Supplementary Table 9).

**Fig. 4:**
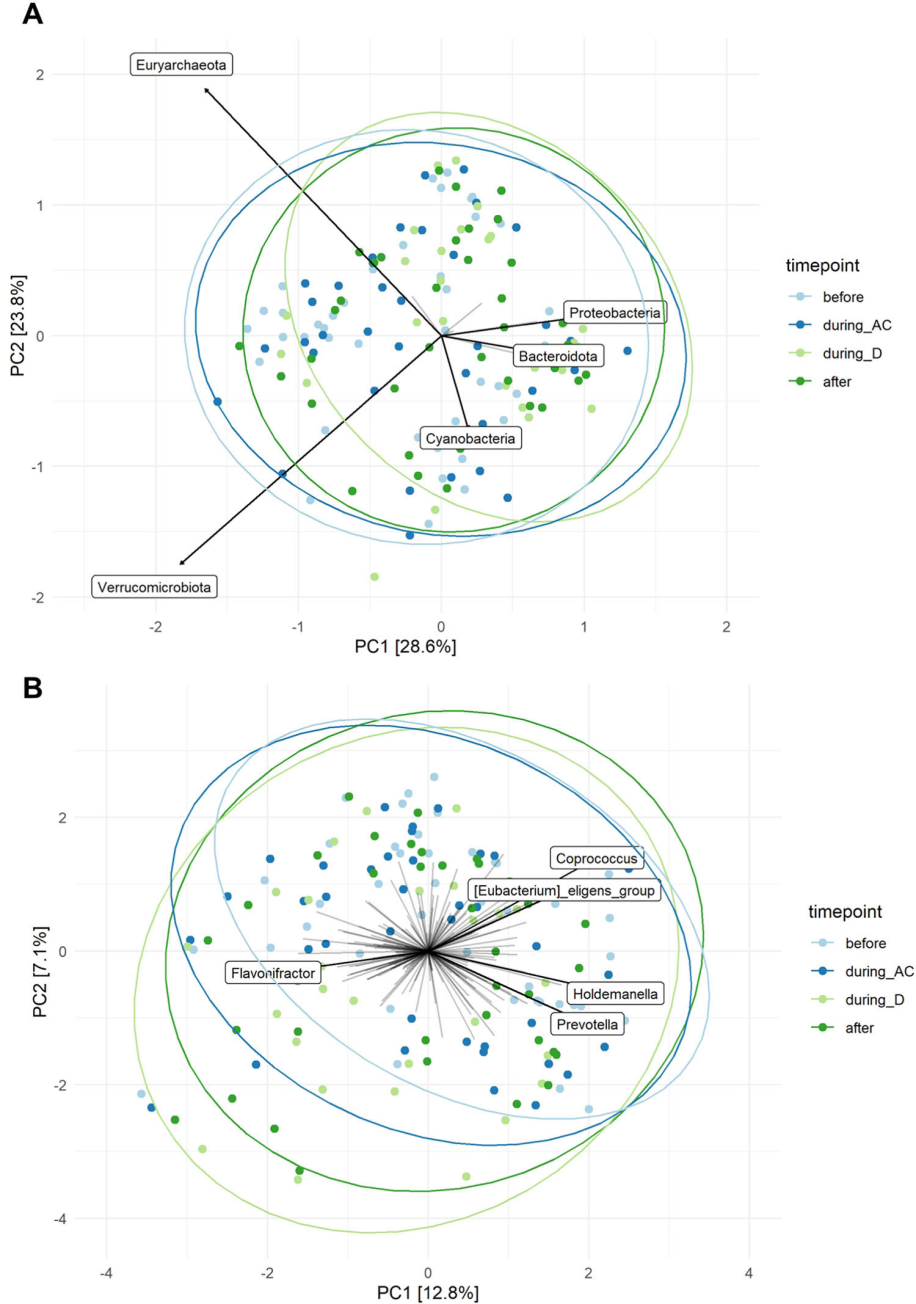
Ordination plots. Ordination plots derived from unconstrained Principal Components Analysis (PCA) based on the Aitchison distance, showing the overall composition of the microbial community at phylum (**A**) and genus level (**B**) before AC-D (*n* = 44), during AC (*n* = 43), during D (*n* = 29) and after AC-D treatment (*n* = 37). Taxa that were present in less than five samples were excluded from this analysis. Data were transformed using centre-log-ratio transformation. Names are given for taxa, which contributed most to overall microbial variation.

Principal component analysis (PCA) showed large heterogeneity in individual microbial community structures. Permutational analysis of variance (PERMANOVA) revealed that there was no statistically significant association between overall microbial community structure at phylum (*p* = 0.086) and genus (*p* = 0.102) level and the different sampling time points (Fig. 4). In line with these PERMANOVA results, dbRDA indicated no effect of the sampling timepoint on microbial community structure after partial out the effect of patient ID (genus level: variance = 2.0006, *p* = 1.0, phylum level: variance = 2.2747, *p* = 1.0).

Furthermore, we identified no consistent significant differences in microbial community structure between patients with or without cumulative therapeutic and prophylactic antibiotic use before or during AC-D (Supplementary Figure 1).

**Fig. 5:**
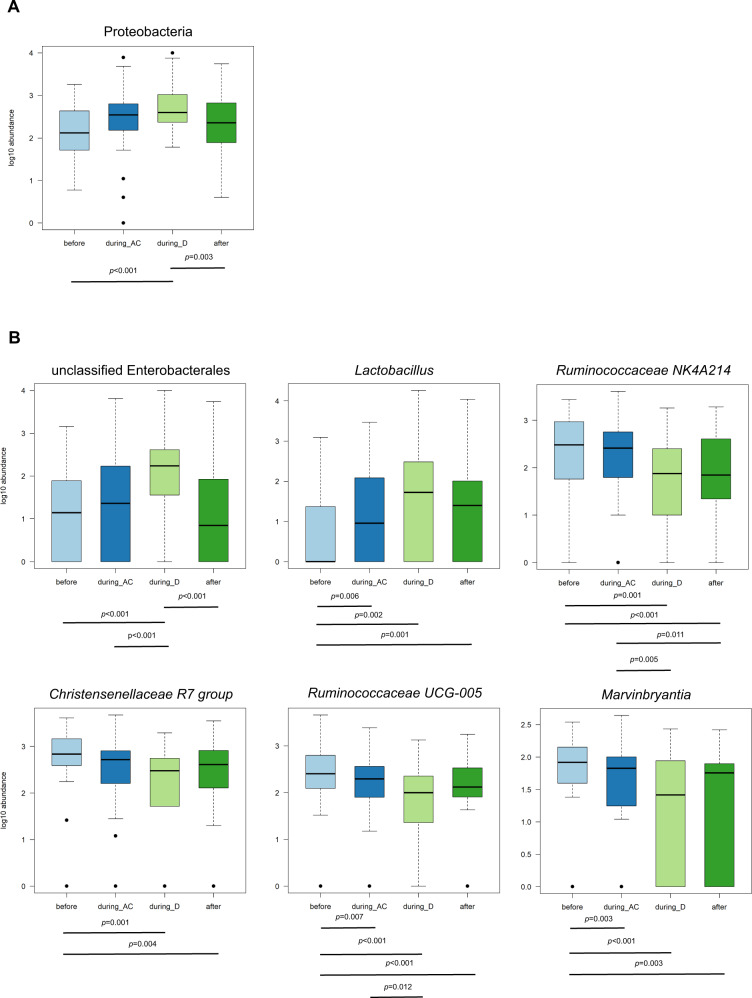
Differential abundant taxa during the course of AC-D. Log^10^ abundance of taxa with significant differential abundance before AC-D (*n* = 44), during AC (*n* = 43), during D (*n* = 29) and after AC-D treatment (*n* = 37). *P* values below boxplots indicate significant differential abundances analysed with a pairwise Wilcoxon signed-rank sum test (Supplementary Table 10). **A** Phylum level. **B** Genus level. The boxplots show the medians, IQRs, minimum, maximum and outliers.

At phylum level, ANCOM-II analysis identified that Proteobacteria were differently abundant during the course of AC-D (Fig. 5A). This significant change over time was confirmed by Friedman’s analysis of variance (ANOVA) (*p* = 0.006). More specifically, pairwise comparison indicated that the abundance of Proteobacteria increased during D, and decreased after AC-D treatment.

Furthermore, according to differential abundance analysis using ANCOM-II, eight genera were differently abundant during the course of AC-D (Fig. 5B). Except for *Turicibacter* and *Intestinibacter*, Friedman’s ANOVA using log^10^(1 + *x*) abundance confirmed these results and indicated significant changes for unclassified Enterobacterales (*p* < 0.001), *Lactobacillus* (*p* = 0.004), *Ruminococcaceae NK4A214 group* (*p* < 0.001), *Marvinbryantia* (*p* = 0.020), *Christensenellaceae R7 group* (*p* = 0.008) and *Ruminococcaceae UCG-005* (*p* < 0.001).

Unclassified Enterobacterales and *Lactobacillus* increased during AC-D treatment. After AC-D treatment, unclassified Enterobacterales decreased (*p* < 0.001). Abundances of the *Ruminococcaceae NK4A214 group*, the *Christensenellaceae R7 group*, *Ruminococcaceae UCG-005* and *Marvinbryantia* decreased during AC-D treatment (Fig. 5 and Supplementary Table 10). More information on longitudinal changes in bacterial abundances in individual patients can be found in Supplementary Figures 2 and 3.

Of note, patients who received antibiotics between T0 and T1 had significantly lower levels of *Christensenellaceae R7 group* at T1 (*p* = 0.001). Furthermore, patients who received antibiotics between T1 and T2 had significantly lower levels of *Marvinbryantia* at T2 (*p* = 0.028).

### Associations of microbiota richness, diversity and composition with chemotherapy toxicity

The most common Common Terminology Criteria for Adverse Events (CTCAE) toxicities are reported in Supplementary Figure 4 and Supplementary Tables 11 and 12. During docetaxel (T2), 19% experienced grade 1 diarrhoea and 19% grade 2. Observed species richness and Shannon index at T2, as well as T3, were negatively correlated with diarrhoea at T2. Patients with any grade diarrhoea during D (T2) had a significantly lower level of observed species richness at T2 compared to patients without diarrhoea (*p* = 0.039). Patients with any grade diarrhoea after AC-D treatment (T3) had a lower Shannon index at T3 compared to patients without diarrhoea (*p* = 0.006). Diarrhoea at T3 was negatively correlated with the levels of *Ruminococcaceae UCG-005 (p* = 0.027*) and the Ruminococcaceae NK4A214 group (p* = 0.033*)*. Nausea at T3 was negatively correlated with observed species richness (*p* = 0.048) and Shannon index (*p* = 0.029). There were no correlations between oral mucositis, hand-foot syndrome or peripheral sensory neuropathy with observed species richness or Shannon index at the different time points.

PERMANOVA showed that microbial community structure on both phylum and genus levels during AC (T1) and during D (T2) was not associated with diarrhoea, nausea, oral mucositis, hand-foot syndrome or peripheral sensory neuropathy (Supplementary Table 13).

Diarrhoea was not correlated to previous therapeutic antibiotics, perioperative prophylactic antibiotic administration, or antibiotic exposure during the course of AC-D treatment.

### Associations between pathologic response and intestinal microbiota richness, diversity and composition in patients treated with neoadjuvant AC-D

In total, 18 patients received neoadjuvant chemotherapy. The clinical characteristics of the neoadjuvant subgroup are presented in Supplementary Tables 14–16.

Response measured after AC-D according to EUSOMA could not be determined in one patient with occult breast cancer (cTxN2). After AC-D treatment one patient (6%) achieved pathologic complete response (pCR), six patients (35%) presented with <10% remaining tumour cells, four patients (24%) with 10–50% remaining tumour cells and six patients (35%) with >50% remaining tumour cells. Accordingly, ten patients were classified as low-responders and seven as high responders (Supplementary Table 17). Baseline characteristics were not different between low and high responders (Supplementary Table 18). No differences in clinical characteristics were observed between low and high responders after AC-D (Supplementary Table 19). Before AC-D, during AC, during D and after AC-D, both α-diversity measures were not significantly different between low and high responders (Supplementary Table 20). PERMANOVA revealed that there was no statistically significant association between baseline microbial community structure and response after AC-D at phylum (*p* = 0.073) and genus (*p* = 0.130) levels. There were no differences in bacterial abundances at baseline between low and high responders.

### Influence of surgery and perioperative use of prophylactic antibiotics

In order to address the potential confounding effects of breast cancer surgery and the perioperative use of prophylactic antibiotics, we also analysed intestinal microbiota composition in the adjuvant (*n* = 26) and neoadjuvant group (*n* = 18) separately. In contrast to the adjuvant group, patients treated in the neoadjuvant setting did not receive surgery and/or prophylactic antibiotics yet.

Baseline α-diversity measures were not significantly different between patients receiving neoadjuvant or adjuvant chemotherapy (observed species richness: *p* = 0.543; Shannon index: *p* = 0.254). PCA and PERMANOVA showed that microbial community structure was significantly different between patients treated in the adjuvant or neoadjuvant setting respectively (*p* = 0.037 on phylum level and *p* = 0.048 on genus level, Supplementary Figure 5). However, ANCOM-II analysis showed that only the abundance of the genus *Dialister* was found to be higher in adjuvant patients (Supplementary Figure 6).

In addition, we examined whether the longitudinal changes in bacterial abundances that were identified in the whole group, could be replicated when analysing neoadjuvant and adjuvant patients separately. Differential abundance analysis using the ANCOM-II workflow, confirmed significant changes in the abundance of Proteobacteria, unclassified Enterobacterales and *Lactobacillus* during AC-D in the neoadjuvant group, while the other taxa did not change significantly. In the adjuvant group, similar to the neoadjuvant group, the abundance of unclassified Enterobacterales changed significantly during AC-D. In addition, other genera (*Intestinibacter, Clostridium sensu stricto, Turicibacter*) also changed in abundance during AC-D in the adjuvant group.

As described earlier, 46% (*n* = 12) of the adjuvant-treated patients received perioperative prophylactic antibiotics before inclusion, while neoadjuvant patients were not treated with prophylactic antibiotics. Therefore, the influence of prophylactic antibiotic administration was investigated in more detail within the group of adjuvant-treated patients. Baseline α-diversity measures were not significantly different between adjuvant-treated patients with or without perioperative prophylactic antibiotic administration (observed species richness: *p* = 0.667, Shannon index: *p* = 0.155, Supplementary Table 7B). PCA and PERMANOVA indicated that baseline microbial community structure was associated with perioperative use of prophylactic antibiotics on genus level (*p* = 0.026), but not on phylum level (*p* = 0.838, Supplementary Figure 5).

## Discussion

This longitudinal pilot study examined the associations between adriamycin, cyclophosphamide and docetaxel (AC-D) treatment and intestinal microbiota, as well as the associations between the intestinal microbiota, chemotherapy toxicity and tumour response in ER+ and HER2− postmenopausal breast cancer patients. Our study showed that during AC-D treatment observed species richness was reduced and the abundance of specific microbial taxa changed. In addition, diarrhoea was associated with lower α-diversity. In addition, we did not detect associations between pathologic response and baseline microbiota richness, diversity and composition in a small group of neoadjuvant-treated patients.

Concerning the observed changes in microbiota richness, diversity and composition in postmenopausal breast cancer patients, no comparable longitudinal clinical studies during AC-D treatment are available. To our knowledge, only the studies of Yulzari et al.^18^ and Terrisse et al.^19^ are comparable to our study exploring the role of intestinal microbiota in breast cancer patients treated with chemotherapy. Yulzari et al.^18^ collected faecal samples of 28 breast cancer patients prior to the start of (neo)adjuvant adriamycin, cyclophosphamide and paclitaxel (P) to study metabolic changes during chemotherapy. However, they did not analyse longitudinal microbiota changes.

In the context of α-diversity, our results show that observed species richness significantly reduced during the course of AC-D with the lowest levels one month after the last docetaxel administration. Previous studies by Montassier et al.^20^ and Galloway-Peña et al.^21^, in patients with acute myeloid leukaemia or Non-Hodgkin lymphoma respectively, observed similar α-diversity reductions during different chemotherapy regimens. In general, a lower microbial α-diversity is associated with diseases of metabolic and immunologic origin^22^. As a next step, the consequences of reduced α-diversity warrant further investigation, for example by studying microbial functions and long-term clinical associations between dysbiosis, chemotherapy toxicity, tumour response or recurrence-free survival.

Besides the reduction of α-diversity, the abundance of specific microbial taxa changed during the course of AC-D treatment. In our small study population, a general trend was observed, in which genera of the *Ruminococcaceae NK4A214 group*, *Christensenellaceae R7 group*, *Ruminococcaceae UCG-005* and *Marvinbryantia* decreased during AC-D and recovered again after AC-D treatment. The abundance of Proteobacteria, unclassified Enterobacterales and *Lactobacillus* significantly increased during AC-D treatment. After AC-D treatment, Proteobacteria and unclassified Enterobacterales significantly decreased, reaching levels comparable to baseline.

Our results suggest that the *Ruminococcaceae NK4A214 group*, *Christensenellaceae R7 group*, *Ruminococcaceae UCG-005* and *Marvinbryantia* might be more sensitive to the effect of AC-D. Many bacteria within the family of Ruminococcaceae are able to produce short-chain fatty acids (SCFA) by degrading polysaccharides. SCFA positively influence intestinal homoeostasis and is known to be involved in immunologic and metabolic functions^23,24^. Therefore, reduction of these bacteria during chemotherapy might contribute to the manifestation of intestinal inflammation and dysregulated homoeostasis.

In contrast to the genera that decreased during AC-D, Proteobacteria and specifically unclassified Enterobacterales, increased during chemotherapy and decreased after AC-D treatment. This could be explained by AC-D-induced intestinal inflammation in combination with the facultative anaerobic properties of these bacteria. It has been demonstrated that cyclophosphamide and adriamycin are able to disrupt and impair the intestinal barrier, which resulted in the translocation of bacteria via the intestinal wall causing systemic inflammation in mice^13,14,25,26^. In addition, the Enterobacterales order includes familiar pathogens such as *Salmonella, Escherichia coli* and *Shigella*, which are known to be associated with intestinal inflammation. Furthermore, Enterobacterales includes facultative anaerobic bacteria, which means that these bacteria have a growth advantage when the blood flow increases due to intestinal inflammation. This systemic inflammation, accompanied by higher blood flow, may contribute to increased levels of unclassified Enterobacterales and its phylum, Proteobacteria. Subsequently, the bloom of Proteobacteria/Enterobacterales at the expense of genera from the Ruminococcaceae family might promote further intestinal inflammation during AC-D.

The largest differences in differentially abundant taxa were observed during docetaxel treatment. Previous research has demonstrated that docetaxel and its metabolites are mainly (75%) eliminated via the faeces, while cyclophosphamide is mainly excreted via the kidneys (up to 70%)^27,28^. Due to high exposure of the gastrointestinal tract to docetaxel and its metabolites, we hypothesise that the direct effect of docetaxel on the intestinal microbiota is more evident compared to the direct effect of cyclophosphamide. As discussed above, it is expected that cyclophosphamide might have a more indirect immune-mediated effect on the intestinal microbiota^9,15^.

Recently, Terrisse et al. (2021) published a comparable French study, where the intestinal microbiota of 63 patients who received (neo)adjuvant eight cycles of anthracycline or anthracycline-taxane-based therapy were analysed with metagenomic shotgun sequencing^29^. Compared to baseline, richness increased after chemotherapy, which was in contrast with our study, where observed species richness significantly decreased after chemotherapy. Furthermore, the study by Terrisse et al. observed at the species level that chemotherapy increased the abundance of *Methanobrevibacter smithii*, *Dorea formicigenerans* and *Ruminococcus torques* and that chemotherapy tended to reduce the species of *Clostridium asparagiforme, Bacteroides uniformis* and *Eggerthella lenta*. We were not able to identify these microbiota shifts at the species level in our study, because taxa were annotated at genus level. However, the shifts described by Terrisse et al. do not correspond with our findings on genus level, where *Ruminococcaceae UCG-005 and Ruminococcaceae NK4A214* decreased after chemotherapy. These discrepancies could be due to some methodological differences between the two studies, limiting comparability. First of all, in the French study patients were not homogenous concerning tumour subtype and systemic cancer therapy scheme. In the French study, up to 24% of the breast cancer patients had triple-negative breast cancer, while in our study only ER+ patients were included. 60% of the French study patients received additional endocrine therapy before the last faecal sample collection, and 31% of the patients received HER2-directed therapy. In addition, sampling time points were different with samples before and after chemotherapy in the study of Terrisse et al. and four sampling time points in our study. With respect to the potential interaction between the oestrogen metabolism and the intestinal microbiota via microbial β-glucuronidase, no distinction was made between pre- or postmenopausal women in the French study, while we only included postmenopausal women. In line with this, Zhu et al.^30^ indicated microbial differences between postmenopausal breast cancer patients and postmenopausal controls but not between premenopausal breast cancer patients and premenopausal controls, potentially indicating that the intestinal microbiota behaves differently in postmenopausal breast cancer patients. Despite these differences in methodological design, these two small studies together form an important basis for further research in this field.

Concerning chemotherapy toxicity, we detected that patients with any grade of diarrhoea during docetaxel treatment had significantly lower observed species richness compared to patients without diarrhoea. Furthermore, diarrhoea was not correlated to antibiotic use prior to AC-D treatment or during AC-D treatment. This makes the assumption stronger that patients suffered from AC-D-induced diarrhoea. In addition, lower performance scores, as well as increased toxicity levels during AC-D treatment, further confirm the systemic inflammatory effects of AC-D treatment. Limited clinical studies confirmed a decrease in microbial richness and its association with diarrhoea in patients undergoing chemotherapy^31^. It might be speculated that patients with lower microbial richness have a higher risk to develop diarrhoea or vice versa. However, the exact mechanism by which AC-D-induced diarrhoea occurs should be examined further, for example using the TIMER (translocation, immunomodulation, metabolism, enzymatic degradation and reduced diversity) model that was recently proposed by Alexander et al. (2017)^32^.

In our small group of neoadjuvant-treated patients, we did not detect associations between pathologic response and baseline intestinal microbiota richness, diversity and composition. This is in contrast to the results from Terrisse et al.^19^, who explored the associations between anthracycline, taxane-based and/or hormone therapy and the intestinal microbiota in breast cancer patients. In both pre and post-chemotherapy faecal samples specific microbiota was associated with either a worse prognosis (lymph node-positive patients and TNM staging >1) or a more favourable prognosis (lymph node-negative patients and/or TNM stage 1). Goubet et al.^11^ observed a longer survival in patients with non-small cell lung cancer and ovarian cancer with an *Enterococcus hirae* and *Barnesiella intestinihominis* specific interferon gamma-mediated tumour response. Comparability between our study and the study of Goubet et al. (2018) is limited since species-level differences could not be analysed in our study. In addition, it concerns other cancer types and the effects of adriamycin and docetaxel were not taken into account by Goubet et al. Furthermore, our results were based on a relatively small sample size of 18 patients. Therefore, these observations should be interpreted carefully and warrant further investigation in a larger study population. In addition, since the pathologic response to neoadjuvant chemotherapy is not a useful surrogate endpoint in ER+, HER2− breast cancer^33^, evaluating the recurrence-free survival of these patients would be of interest in future research into associations between the intestinal microbiota and treatment efficacy.

In the present cohort, patients were included in both the adjuvant and neoadjuvant settings. Although the group of neoadjuvant patients was relatively small in our cohort, we could confirm the observed significant increase in the abundance of Proteobacteria, unclassified Enterobacterales and *Lactobacillus* during the course of AC-D treatment in the neoadjuvant group. However, we observed some clinical differences between adjuvant and neoadjuvant patients, for instance, higher clinical tumour stages and higher Karnofsky Performance Scores in neoadjuvant patients. In addition, analysis of the intestinal microbiota indicated differences in microbiota community structure between these groups. Longitudinal changes in abundance, which were observed in the whole group, could not be confirmed in both subgroups. Consequently, we cannot rule out that breast cancer surgery in the adjuvant group might have a confounding effect on our results. Due to these potential confounding factors, it is likely that the intestinal microbiota behaves differentially in these two subgroups, despite the fact that patients receive the same AC-D treatment. Therefore, it would be beneficial to perform similar future studies in neoadjuvant patients only, in order to exclude breast cancer surgery as a confounding factor. However, the fact that the abundance of unclassified Enterobacterales changed consistently among all groups, does suggest that overgrowth of these bacteria could be a common phenomenon during chemotherapy and requires further evaluation.

Next to breast cancer surgery, it is widely described that antibiotic exposure can disturb the intestinal microbiota. For this purpose, additional in-depth analyses were performed to examine the potential influence of antibiotic administration on our results. The effect of antibiotic administration in the total group was mainly observed in adjuvant patients receiving perioperative prophylactic antibiotics. Therefore, we also analysed the influence of perioperative administration of prophylactic antibiotics on intestinal microbiota composition and diversity, and we identified significant effects on baseline microbial community structure. As a consequence, perioperative prophylactic antibiotic administration might have a confounding effect on our results. As described above, this could be prevented in future studies by including only patients receiving neoadjuvant chemotherapy. Of note, cumulative antibiotic use (therapeutic or prophylactic) before or during AC-D did not indicate differences in microbiota composition at T3. This could be partly explained by time and the interaction with other microbiota-modulating factors such as docetaxel treatment. Consequently, the primary observed prophylactic antibiotic effect might diminish or disappear over time^34^. In addition, as discussed earlier, the direct effect of docetaxel on the intestinal microbiota^27^ might be more evident compared to the effect of (earlier) prophylactic antibiotic administration. None of the patients used prebiotics, probiotics or nutritional supportive drinks during the course of AC-D treatment. This means that the differences in microbiota richness, diversity and composition are not attributable to these microbiota-modulating agents.

There are several limitations and strengths of this study. The main limitation is the small sample size. It is not possible to draw conclusions concerning causal relationships or to predict therapy outcomes, based on this small cohort. Consequently, the current study should be seen as a pilot study providing insights into the feasibility of a study with longitudinal microbiota sampling in patients during AC-D. Our results provide early indications that there might be an interaction between the intestinal microbiota and AC-D. Furthermore, our results indicate that patients treated in the adjuvant or neoadjuvant setting should be analysed separately in future studies.

In addition, another limitation is the use of pCR, as it is known that pCR is not a useful surrogate endpoint for the specific breast cancer subtype studied (ER+/HER2−)^33^. Besides that, it was only possible to conduct chemotherapy response measurements in the subgroup of neoadjuvant-treated patients. As a consequence, the group size was reduced from 44 to 18 patients. In addition, response measurement based on the residual tumour is not possible in adjuvant-treated patients, since adjuvant patients will be subjected to tumour resection first. To circumvent this, increased sample sizes and other response measurements should be used, for instance, recurrence-free survival or progression-free survival^35^.

Unfortunately, no analysis of bacterial metabolites (e.g., SCFA), has been performed. Insights into the levels of SCFA would provide more knowledge on the intestinal bacterial activities involved in the regulation of the host’s immune system and metabolism, as well as their associations with cancer treatment^36^. Besides that, co-medication, diet and surgery-related factors, such as type, route and duration of antibiotic administration, might form alternative explanations for our findings.

Lastly, sequencing of the V4 region rather than larger segments (e.g., V3–V4 regions) has some limitations, but also advantages. Although regions such as V3–V4 span a longer segment of the 16 S rRNA gene, the overlap between forward and reverse reads are shorter, and in particular with amplicon sequence variant calling this has been shown to result in spurious inflation of the amplicon sequence variant (ASV) diversity^37^. With 250 bp paired-end sequencing, the overlap between forward and reverse reads is (near to) complete when sequencing the V4 region and consequently the sequencing errors are significantly reduced. Moreover, in contrast to what might be expected based on the longer region, a recent extensive comparison showed that analysis of the V3–V4 results in a less accurate taxonomic assignment when compared to the V4 region^37^. However, in silico analyses showed that short-read sequencing of hypervariable regions cannot achieve the same level of taxonomic resolution as can be achieved by sequencing of the entire 16 S rRNA gene^38^. Full-length 16 S rRNA gene sequencing allows better discrimination between closely related species^39^. To sequence even longer regions other platforms such as MinION nanopore or PacBio have recently been used. However, these platforms generate read data with significantly lower nucleotide accuracy than the Illumina platform due to random base-calling errors. This has recently been overcome by a novel technology developed by Loop Genomics, which enables long-read sequencing by utilising an existing Illumina short-read sequencer combined with a unique molecule barcoding technology. This method was not yet widely applied at the time of initiating our lab analyses^40^.

One unique advantage of this study is its relatively homogenous study population. To make the group as homogeneous as possible, we only included postmenopausal women to exclude the effect of physiologically higher oestrogen levels in premenopausal patients. To exclude the effect of HER2-targeted therapy, HER2 receptor-positive patients were not included. Based on the expected differences between the groups described above, our results are not directly generalisable to premenopausal patients, HER2+, or triple-negative breast cancer patients. Another strength of this study is the longitudinal design, including the collection of faecal samples at four different time points. In addition, in-depth analyses of antibiotic administration have been performed to reveal potential confounding effects of antibiotic administration.

As described above, our study might be seen as a pilot study, providing guidance for the design of future studies in this field of research. Future research could focus on the role and function of the bacteria that increased or decreased during chemotherapy treatment. This could be done with a quantitative assessment of microbial metabolites. Also, full-length 16 S rRNA gene sequencing or high-throughput whole metagenomic shotgun sequencing, including the possibility to determine bacterial metabolic capacity will be highly relevant to further establish the microbiota composition, including its microbial functions and their associations with chemotherapy toxicity. In addition, to overcome the influence of small sample size on tumour response measurement in the neoadjuvant treated group, larger breast cancer cohorts should be recruited. Finally, it will be highly relevant to compare our results with upcoming studies that address the link between intestinal microbiota and chemotherapy in breast cancer patients (e.g., NCT03586297 and NCT04138979) to see whether the chemotherapy-induced patterns are similar among different breast cancer subtypes and if it is possible to identify key species susceptible to chemotherapy.

In conclusion, this is the first clinical study with longitudinal faecal sampling in breast cancer patients that explored the associations between adriamycin, cyclophosphamide and docetaxel treatment and the intestinal microbiota, as well as the impact of the intestinal microbiota on chemotherapy toxicity and tumour response in ER+ and HER2− postmenopausal breast cancer patients. We reported shifts in intestinal microbiota richness and composition during AC-D treatment. Our findings provide important insights into an association between chemotherapy and intestinal microbiota in postmenopausal ER+ and HER2− breast cancer patients. Our results emphasise the necessity to further explore chemotherapy-induced microbiota changes and potential metabolic and immunologic consequences in breast cancer patients.

## Methods

### Patients

Between November 2017 and February 2020, breast cancer patients were prospectively enroled in four Dutch hospitals. Eligible patients were postmenopausal women with histologically proven ER+ (≥10%), and human epidermal growth factor receptor-2 (HER2) negative breast cancer^41^ starting with (neo)adjuvant chemotherapy. Exclusion criteria included distant metastasis, previous chemotherapy and therapeutic antibiotics within three months prior to AC-D treatment.

The study was approved by the Medical Ethics Committee azM/UM (METC 17-4-075). The study was conducted in accordance with the Declaration of Helsinki and Good Clinical Practice. Each patient provided written informed consent.

### Treatment

During the study period, patients received four cycles of adriamycin (A), 60 mg/m^2^ i.v. and cyclophosphamide (C) 600 mg/m^2^ i.v. on day 1, in either a two-weekly (dose-dense, dd) or three-weekly cycle. AC treatment was followed by four cycles of docetaxel (D), 100 mg/m^2^ i.v. on day 1, in a three-weekly cycle. Patients received chemotherapy in neoadjuvant or adjuvant settings according to standard care using the Dutch guideline for systemic breast cancer treatment^42^. These guidelines are largely in line with the ESMO and ASCO guidelines. Corticosteroid administration was provided according to local protocols. In general, 8 mg dexamethasone once a day was provided during each adriamycin/cyclophosphamide cycle on day 1 (i.v.), day 2 (oral) and day 3 (oral). During each docetaxel cycle, 8 mg oral dexamethasone was provided twice a day on the day before, during and after the first day of the cycle. These administrations were uniformly done across all the patients.

### Faecal sample and data collection

Patients collected a faecal sample and completed a questionnaire at four time points: before the start of AC-D (T0), during the second week of the fourth cycle AC (T1), during the second week of the fourth cycle D (T2) and 1 month after the last dose D (T3) (Supplementary Figure 7). Samples were immediately stored in the freezer and transported to the hospital in a cooled container (Sarstedt)^43^. In the hospital, samples were stored immediately at −20 °C and subsequently at −80 °C for long-term storage. Patient characteristics were registered, including chemotherapy dose reductions, prophylactic and therapeutic antibiotic use, prebiotic/probiotic use and the use of nutritional supportive drinks. Therapeutic antibiotic treatment included treatment between one year and three months prior to T0 faecal sample collection. Prophylactic cefazolin administration at the start of the breast cancer operation and prophylactic amoxicillin/clavulanic acid administration after the operation was summarised as perioperative prophylactic antibiotic use. Nutritional status was assessed with the Malnutrition Universal Screening Tool (MUST).

### Clinical characteristics assessed by the questionnaires

Questionnaires were taken from the patient at four timepoints:

T0: Before administration of the first chemotherapy treatment

T1: 1–2 weeks after administration of 4th chemotherapy treatment

T2: 1–2 weeks after administration 16th chemotherapy treatment

T3: 4 weeks after administration of 16th and last chemotherapy

Questionnaire T0–T3:

- Birth month/year
- Fill-in date
- Current weight
- Current length
- Malnutrition Universal Screening Tool (MUST) score: based on the following questions:
  - Are you feeling ill at this moment?
  - Do you have a normal appetite?
  - Did you eat bad the past 5 days?
  - Do you think that you could eat bad for more than 5 days?

- Karnofsky Performance Score (KPS): Scale from 0 to 100
  - 0 = deceased
  - 100 = No complaints, no symptoms of illness

- Did you collect faeces today?
  - If no, what date did you collect faeces?

- CTCAE score (Common Terminology Criteria for Adverse Events)
  - Nausea (scale; 0–3)
  - Vomit (scale; 0–4)
  - Inflammation of the mouth (scale; 0–4)
  - Diarrhoea (patients without stoma) (scale; not applicable, 0–4)
  - Diarrhoea (patients with stoma) (scale; not applicable, 0–4)
  - Unintentional weight loss past 3–6 months (scale; 0–3
  - Constipation (scale; 0–4)
  - Fever (scale; 0–4)
  - Changed feeling (deaf, irritation, tingling) (scale; 0–4)
  - Hand-feet complaints (scale; 0–3)
  - Fatigue (scale; 0–3)
  - Hair loss (scale; 0–2)

Additional questions in questionnaire T0:

- Past treatment with chemotherapy (when, name of therapy, number of treatments)
- Antibiotics use past year (when, name, number of days)
- Prednison (steroids) use past year
- Prednison (steroids) use past month
- Use of oral contraception
- Past use of contraception (stop date; month & year, the total amount of years used)
- Use of Intra Uterine Device (IUD) (type)
- Past use of IUD (type, date of removal, total amount of years used)
- Diabetes (type)
- Smoking (years, number of cigarettes in a day)
- Past smoking (years, number of cigarettes in a day, stop date)
- Past abdominal surgery (what type)
- Crohn’s disease
- Colitis Ulcerosa

Additional questions in questionnaire T1–T3:

- How many of the next tablets did you use since filling in the previous questionnaire (before start of chemotherapy):
  - Metoclopramide (primperan) (number of tablets)
  - Granisetron (kytril) (number of tablets)
  - Diarrhoea inhibitors (Imodium/loperamide) (number of tablets)
  - Antibiotics (If yes, name and duration)
  - Prednison/dexamethasone

### Response measurement

In neoadjuvant patients, pathologic tumour response after neoadjuvant AC-D treatment was assessed using the scoring system according to European Society of Breast Cancer Specialists (EUSOMA). High responders were defined as EUSOMA 1 and EUSOMA 2 (i). Low-responders were defined as EUSOMA 2 (ii), EUSOMA 2 (iii) and EUSOMA 3^44^. The complete definition of the EUSOMA scoring system is presented below.

**EUSOMA 1:** Complete pathological response

- (i): no residual carcinoma.
- (ii): no residual invasive carcinoma but DCIS present.

**EUSOMA 2:** Partial response to therapy.

- (i): minimal residual disease/near total effect (e.g., only a few loose tumour cells or tumour cells located in small groups).
- (ii): evidence of response to therapy but with 10–50% of tumour remaining.
- (iii): >50% of tumour cellularity remains evident, when compared to the previous core biopsy sample, although some features of response to therapy are present (e.g., fibrosis).

**EUSOMA 3:** No response: no evidence of response to therapy.

### Chemotherapy toxicity measurement

Toxicity was scored with CTCAE version 4.0^45^. The following aspects were scored: diarrhoea, peripheral sensory neuropathy, hand-foot syndrome, fatigue, nausea, oral mucositis, vomiting, alopecia and constipation. For binary toxicity analysis, patients with toxicity were defined as having toxicity scores ≥grade 1.

### Faecal microbiota analyses

Metagenomic DNA was isolated using the Ambion MagMax^TM^ Total Nucleic Acid Isolation Kit (*Thermo Fisher Scientific*). We performed a manual pre-processing procedure followed by automated nucleic acid purification with the KingFisher FLEX (*Thermo Fisher Scientific*). In more detail, in order to extract metagenomic DNA, 250 mg of the frozen faecal samples was homogenised in phosphate-buffered saline (PBS) and centrifuged for 1 minute at 900 rpm. For cell lysis, a combination of chemical, mechanical and thermal disruption was used. A lysis buffer containing 1 M Tris-HCl, 0.5 M EDTA, 5 M sterile NaCl and SDS (final concentration 4%) was filled into bead tubes of the Ambion MagMax^TM^ Total Nucleic Acid Isolation Kit *(Thermo Fisher Scientific)* and mixed with 175 µl supernatant of faeces in PBS. Mechanical disruption consisted of a bead-beating procedure using the Fastprep™ Homogeniser *(*5.5 ms for 3 × 1min; resting 1 min in between, *MP Biomedicals)*. Samples were subsequently incubated for 15 min at 95 °C with gentle shaking. After centrifugation for five minutes at 11,000 rpm, the supernatant was filled in an Eppendorf tube. Afterwards, the second round of bead beating and incubation was performed and supernatants were pooled and stored at −20 °C until further analysis. 200 µl of the supernatants were introduced into a KingFisher 96-wells deep well plate *(Thermo Fisher Scientific)*, together with a bead mix of the Ambion MagMax^TM^ Total Nucleic Acid Isolation Kit (*Thermo Fisher Scientific*), isopropanol and lysis buffer. Other plates were filled with wash buffers, elution buffer (+RNAse) and 96-tips for DW magnets *(Thermo Fisher Scientific)*. Afterwards, the prepared plates were introduced into the KingFisher system and the DNA isolation was performed according to the manufacturer’s standard protocol *(Thermo Fisher Scientific)*. After removal of the plates from the system, the plate containing purified nucleic acids was incubated for 15 min at 37 °C for degradation of RNA.

Subsequently, the V4 hypervariable region of the 16 S rRNA gene was amplified in triplicate using the 515 F/806 R barcoded primer pair described previously^46^. Pooled amplicons from the triplicate reactions were purified using AMPure XP purification (Agencourt) according to the manufacturer’s instructions and eluted in 25 μl 1× low TE (10 mM Tris-HCl, 0.1 mM EDTA, pH 8.0). Quantification of amplicons was subsequently performed by the Quant-iT PicoGreen dsDNA reagent kit (Invitrogen) using a Victor3 Multilabel Counter (*Perkin Elmer, Waltham, USA*). Amplicons were mixed in equimolar concentrations to ensure equal representation of each sample and sequenced on an Illumina MiSeq instrument (MiSeq Reagent Kit v3, 2 × 300 cycles, 10% PhiX) to generate paired-end reads of 250 bases (∼25,000 reads/sample)^47^. All basic 16 S rRNA gene sequencing statistics are presented in Supplementary Table 21.

Bioinformatic analysis of the sequencing data was performed using R (version 4.0.3)^48^. For the pre-processing, a standardised in-house pipeline using the software package DADA2 was applied^49^. The pre-processing consisted of the following steps: reads filtering, identification of sequencing errors, dereplication and removal of chimeric sequences.

In order to assign taxonomy, the SILVA 138 database and DECIPHER’s IDTAXA algorithm^50^ were used to annotate to the genus level. Data were expressed as ASVs. Decontam was used with the “either” setting, which combines the two statistical methods prevalence and frequency for the identification of contamination in marker-gene and metagenomics data^51^. Contaminant ASVs identified by decontam, were filtered out, together with ASVs present in <5% of all samples and those with a total abundance of less than 0.001%. After filtering, 816 taxa remained in the analysis. The final file was saved in the phyloseq format^52^.

### Statistical analysis of clinical data

Baseline characteristics, longitudinal clinical data, statistical tests for α-diversity measures and abundances of phyla and genera of interest were analysed in IBM SPSS version 26. For continuous data, normality was tested using the Shapiro–Wilk test. Depending on whether the variable was normally distributed or not, an unpaired *t* test or the non-parametric Mann–Whitney *U* test was applied. Levene’s test was used to test for equal variances. For categorical variables, the non-parametric Chi-square test was performed. In the case of low frequencies of binary variables, a Fisher’s exact test was used.

For longitudinal analysis with two-time points of quantitative variables, a paired sample *t* test or the non-parametric Wilcoxon signed-rank sum test was used. For longitudinal analysis with four-time points, repeated measures ANOVA or Friedman’s ANOVA were used for normally and non-normally distributed data, respectively. For repeated measures ANOVA, Greenhouse-Geisser correction was used when the assumption of sphericity was not met.

Longitudinal significant results were subjected to post hoc Wilcoxon signed-rank sum tests with Bonferroni correction. After Bonferroni correction, *p* values below 0.0125 indicated significance.

Spearman’s rho (*r*_s_) correlation coefficient was used to assess the relationship between ordinal and continuous data. Two-tailed tests were used and in general *p* values below 0.05 were considered statistically significant.

### Statistical analysis of intestinal microbiota data

Non-rarefied data were used for diversity analysis. Both α-diversity indices, including observed species richness and Shannon index, which is a measure of microbial diversity, were calculated on ASV level, using the phyloseq package^52^. Testing the assumptions of normality and homogeneity of variance, and subsequent statistical testing was performed as described in the clinical data analysis section.

The R packages, phyloseq^52^, vegan^53^, microbiome^54^, dplyr^55^, ggplot2^56^ and microViz^57^ were used for ordination and visualisation of taxonomic composition. Taxa present in less than five samples were filtered out for all analyses. Unconstrained ordination was performed using PCA based on Aitchison distances at genus and phylum level^57^. Homogeneity of multivariate dispersions was evaluated by means of the microViz package and was similar in all cases. PERMANOVA, by means of the dist_permanova function from the microViz package^57^, was used to analyse longitudinal changes in overall microbiota composition (based on Aitchison distances). Since this analysis does not account for the clustered nature of the data, i.e., the correlated measurements within subjects, we additionally performed a distance-based redundancy analysis using Aitchison distance by means of the cap scale function from the vegan package^27^. Patient ID was defined as a variable to be partially out in order to account for the correlated data. In addition, PERMANOVA was used for cross-sectional analyses to assess the association between diarrhoea, nausea, oral mucositis, hand-foot syndrome and peripheral sensory neuropathy with overall microbiota composition. Within the neoadjuvant subgroup, PERMANOVA was used to analyse the association between treatment response and overall microbiota composition^57^. Differential abundance analysis, investigating changes of individual taxa abundance on phylum and genus level during the course of AC-D treatment, was conducted using the workflow of ANCOM v.2.1 (random intercept model for repeated measures) which accounts for the underlying compositional structure, sparseness of microbiota data and random effects caused by longitudinal data^58^. We set *α* < 0.05 at 70% (W) of comparisons as the threshold for significance. Structural zeros were not considered as differentially abundant taxa. For the purpose of visualisation, bacterial relative abundance was transformed into log^10^(1 + *x*) abundance by means of the microbiome package^54^. Non-parametric tests based on log^10^(1 + *x*) abundance were used to confirm the ANCOM-II results in SPSS.

## Supplementary information

Supplementary Figures and Tables ACD 23 MAY 2022

## Data Availability

Sequencing data were submitted to Qiita and deposited in the European Nucleotide Archive (ENA)^59^. The accession code is ERP136994. Additional data generated during and/or analysed for the current study are available from the corresponding author on reasonable request.
