# Supplementary material for: Changes in intestinal microbiota in postmenopausal oestrogen receptor-positive breast cancer patients treated with (neo)adjuvant chemotherapy
Source: NPJ Breast Cancer. 2022 Jul 29;8:89. doi: 10.1038/s41523-022-00455-5 (PMC9338016; doi:10.1038/s41523-022-00455-5)
Supplement: Supplementary file 1 — Supplementary Figures and Tables ACD 23 MAY 2022 [file 41523_2022_455_MOESM1_ESM.pdf]

Supplementary Figures

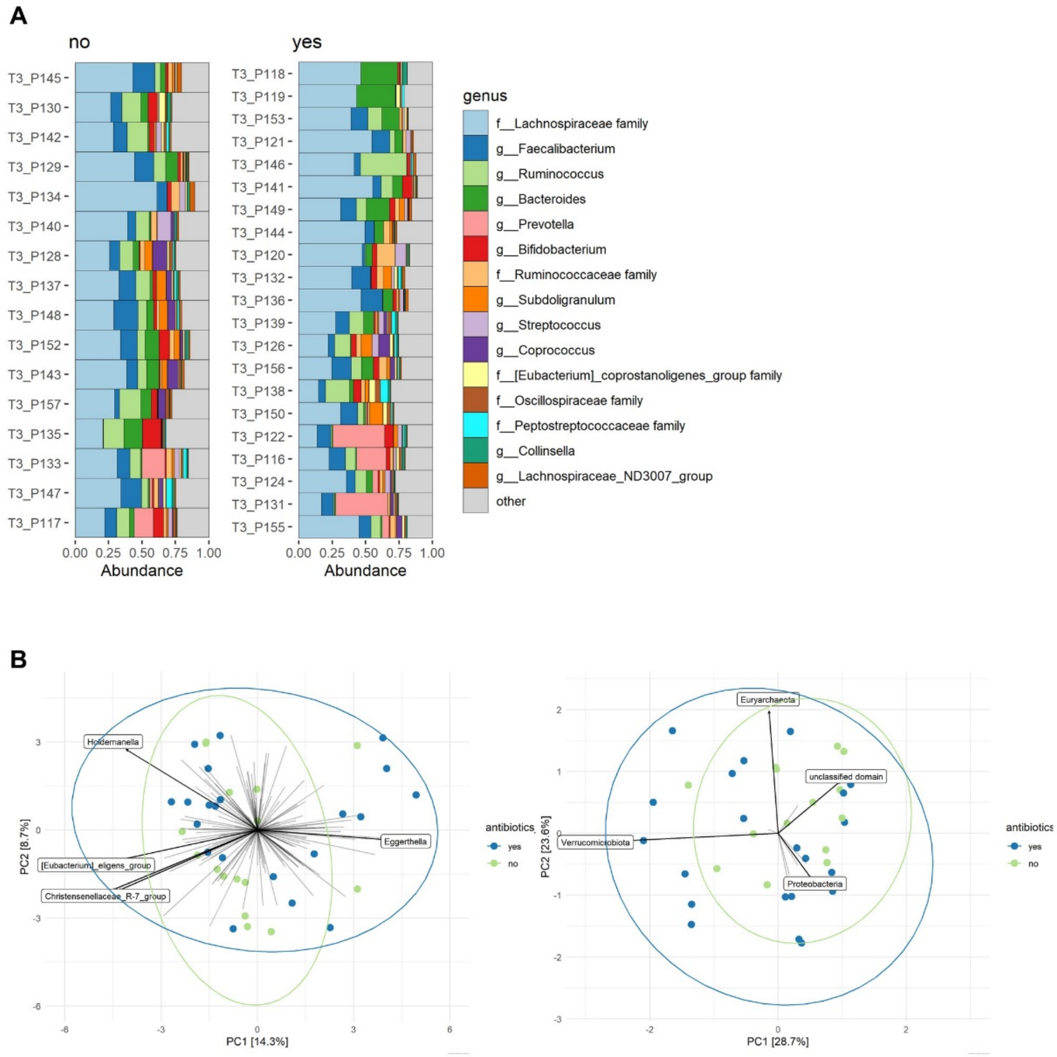

Supplementary Figure 1: A: Relative abundance of the 15 most common genera at sampling timepoint T3 among patients without previous antibiotic use ("no", *n*=16) or with previous antibiotic use before or during AC-D ("yes", *n*=21). **B:** Ordination plots derived from unconstrained Principal Components Analysis (PCA) based on the Aitchison distance, showing differences in intestinal microbiota composition at T3 between patients with (**blue**, *n*=21) or without (**green**, *n*=16) previous antibiotic use before or during AC-D. PERMANOVA showed that there were no statistically significant differences at phylum (*p*=0.280) and genus level (*p*=0.522).

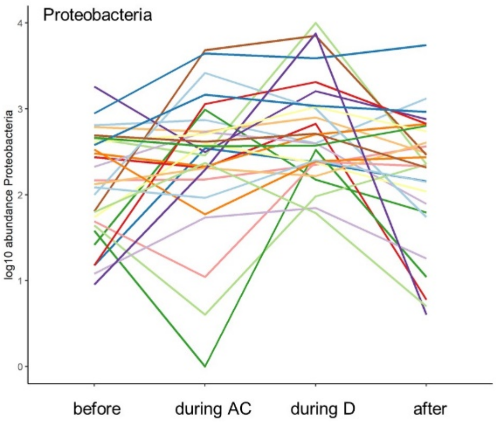

Supplementary Figure 2: Longitudinal changes of Proteobacteria abundance in individual patients. Only complete cases (*n*=28) are displayed. Each line represents an individual patient.

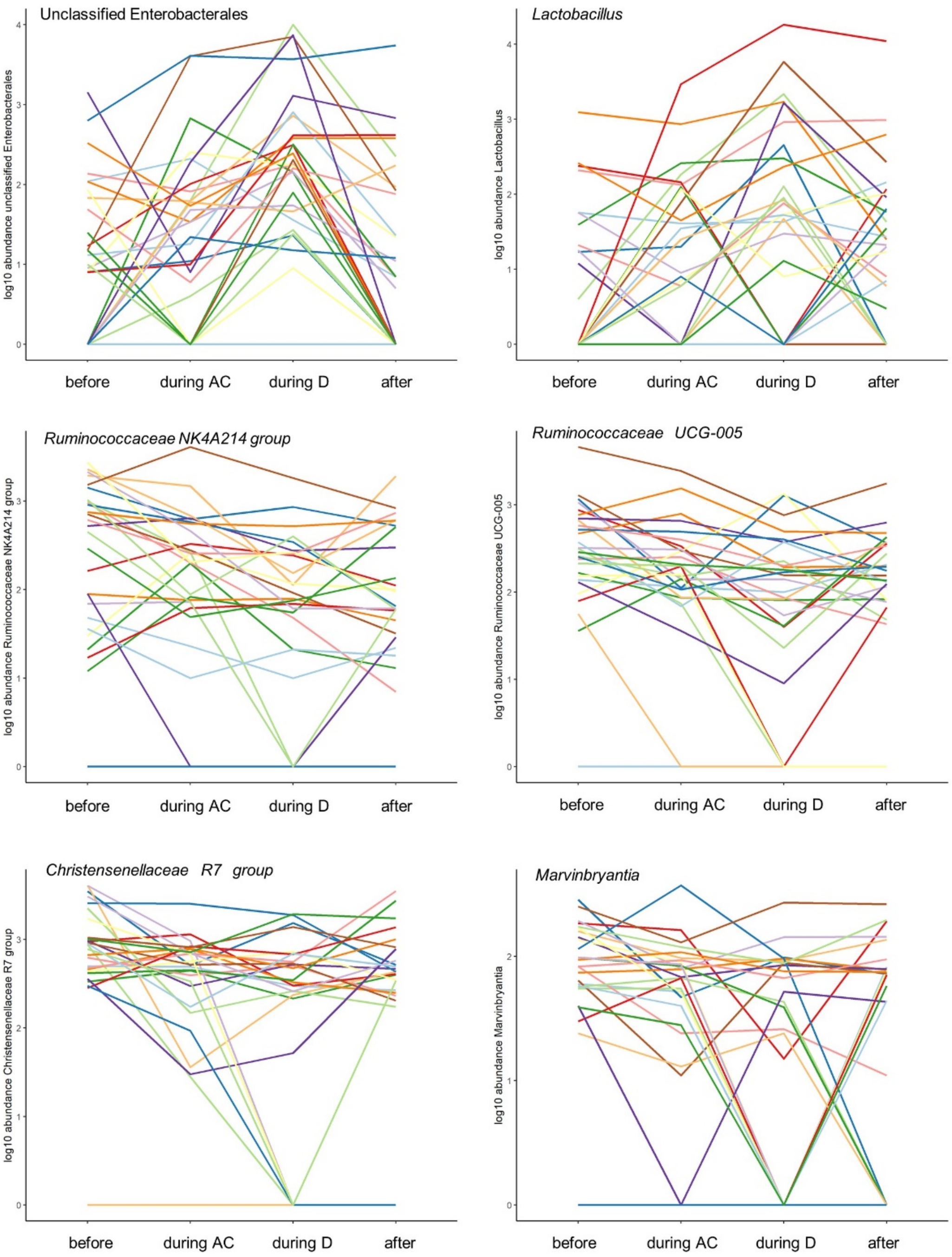

Supplementary Figure 3: Longitudinal changes of abundance of different genera in individual patients. Only complete cases (*n*=28) are displayed. Each line represents an individual patient.

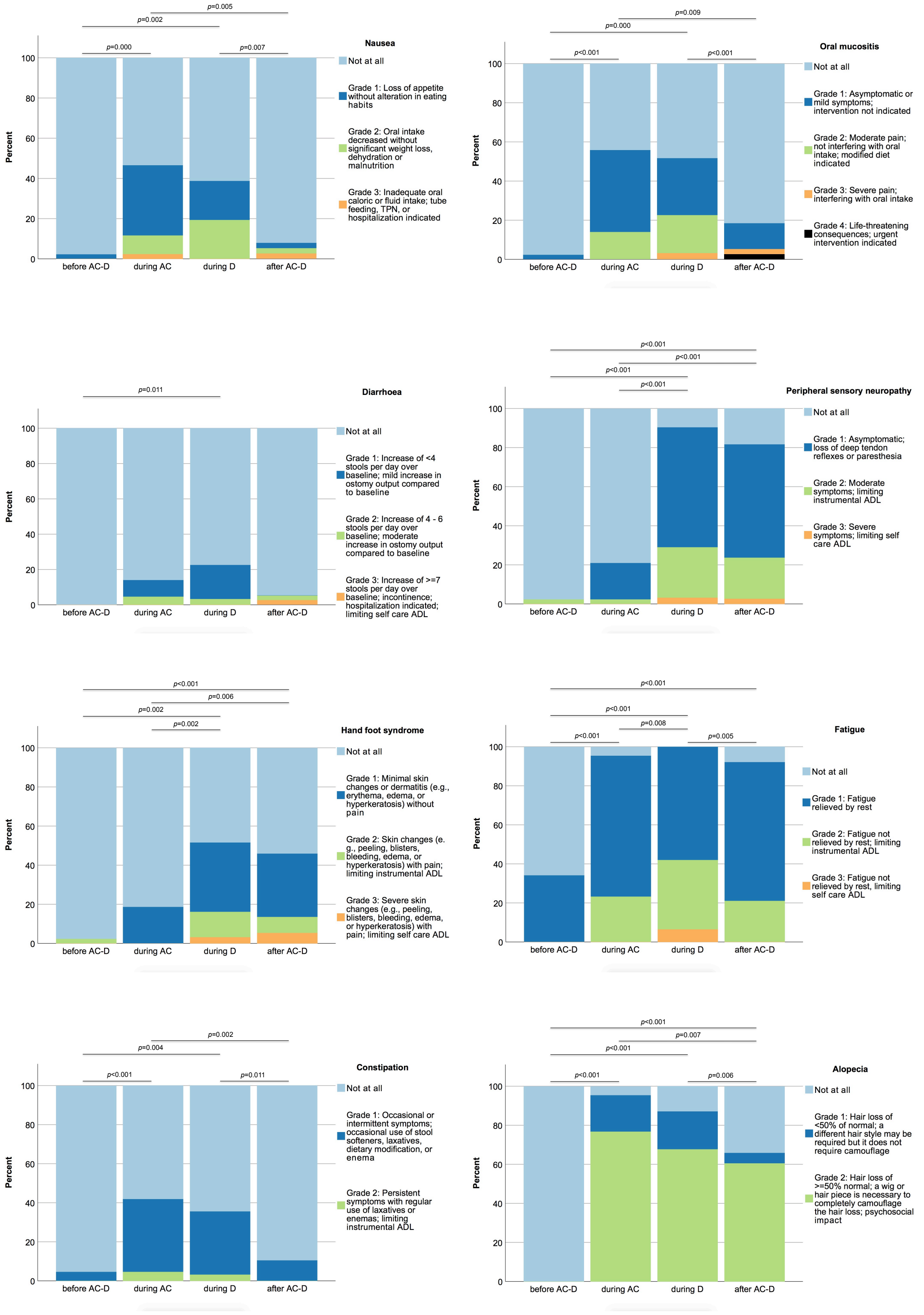

Supplementary Figure 4: Stacked bar charts presenting percentage toxicity grades before AC-D, during AC, during D, and after AC-D. Friedman tests indicated that nausea, oral mucositis, diarrhoea, peripheral sensory neuropathy, hand foot syndrome, fatigue, constipation and alopecia changed significantly over time. Presented *p*-values indicate significant differences between the different time points revealed by the Wilcoxon test with Bonferoni correction (See table S11 for numbers of patients per grade classification).

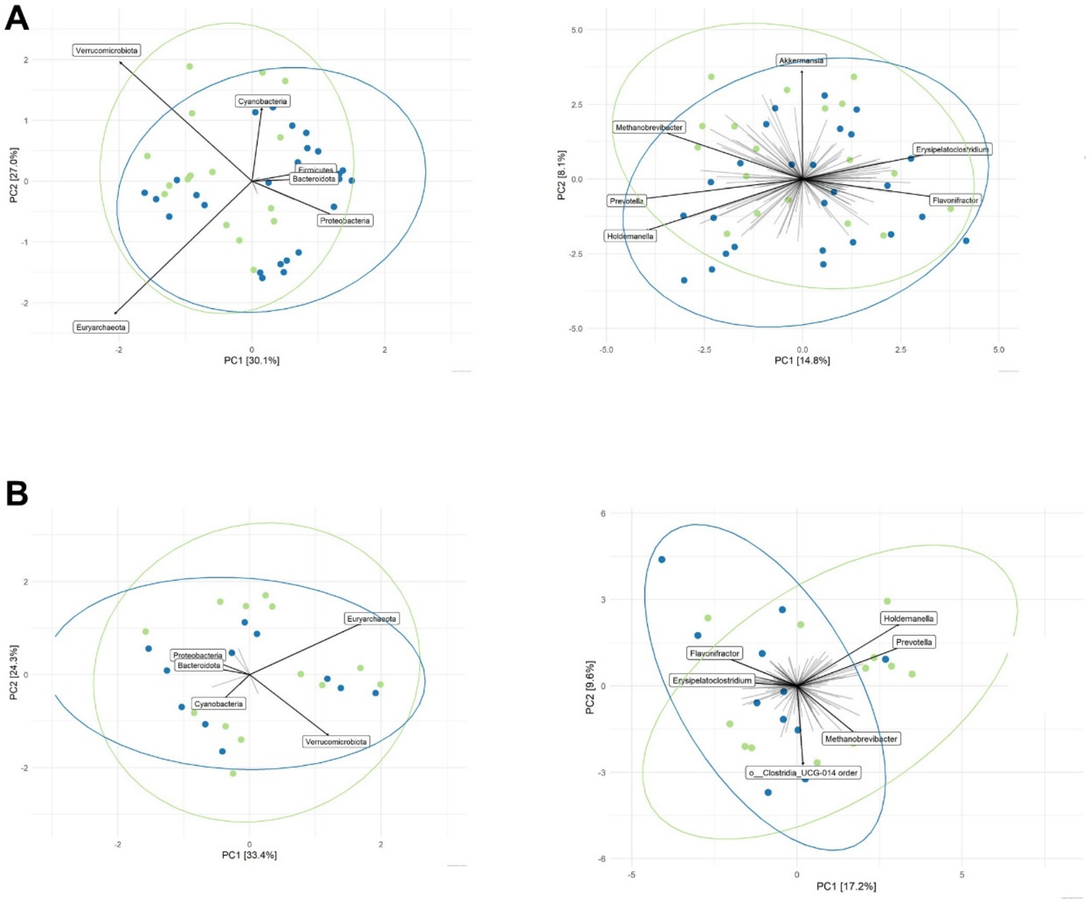

Supplementary Figure 5: A: Ordination plots derived from unconstrained Principal Components Analysis (PCA) based on the Aitchison distance, showing differences in baseline (T0) microbiota composition between adjuvant (**blue**, *n*=26) and neo-adjuvant patients (**green**, *n*=18) at phylum and genus level. **B:** Ordination plots derived from unconstrained Principal Components Analysis (PCA) based on the Aitchison distance at phylum and genus level, showing differences in baseline (T0) microbiota composition between adjuvant patients who received perioperative prophylactic antibiotics (**blue**, *n*=12) and adjuvant patients who did not receive perioperative prophylactic antibiotics (**green**, *n*=14). For all plots, taxa that were present in less than 5 samples were excluded for this analysis. Data were transformed using centre-log-ratio transformation. Names are given for taxa, which contributed most to overall microbial variation.

T0

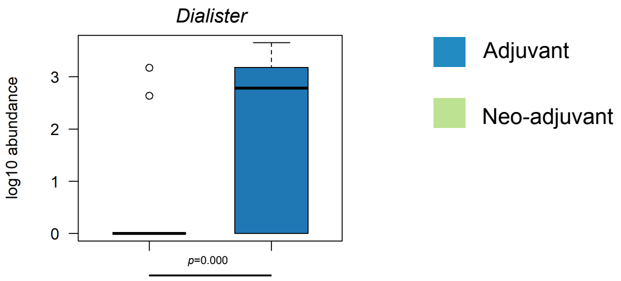

Supplementary Figure 6: Baseline log^{10} abundance of the genus *Dialister* in adjuvant and neo-adjuvant patients. ANCOM-II analysis identified this genus to be differentially abundant among adjuvant (*n*=26) and neo-adjuvant (*n*=18) patients, which was confirmed by a Mann-Whitney-U Test (p<0.001).

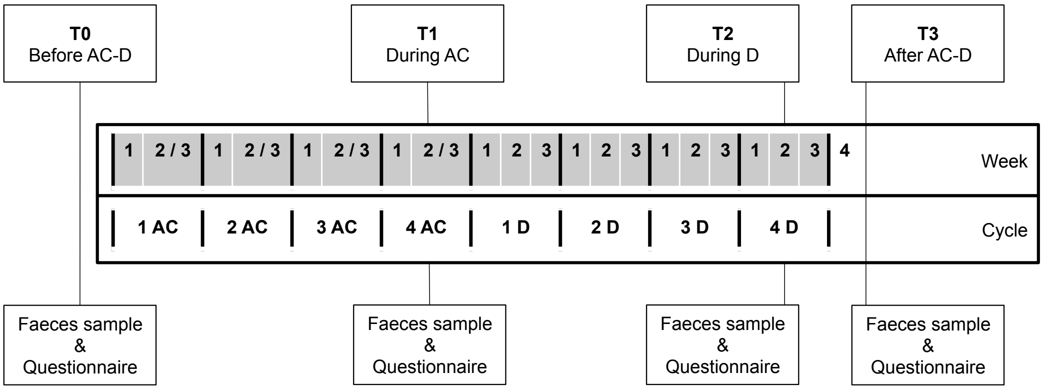

Supplementary Figure 7: Study design.
Patients collected a faecal sample and completed a questionnaire at four time points: **T0** was collected before the start of the AC-D, **T1** during week 2 of cycle 4 AC, **T2** during week 2 of cycle 4 D, and **T3** one month after the dose D. During the study period, patients received four cycles adriamycin (A), 60 mg/m^2 i.v. and cyclophosphamide (C) 600 mg/m^2 i.v. on day 1, in either a two-weekly (dose dense, dd) or three-weekly cycle. AC was followed by four cycles of docetaxel (D), 100 mg/m^2 i.v. on day 1, in a three-weekly cycle.

Supplementary Tables

Supplementary Table 1: Clinical characteristics of the total study population at baseline including the comparison between adjuvant and neoadjuvant treated patients.

**Baseline characteristics**	**Total** (*n*=44)	**Adjuvant** (*n*=26)	**Neoadjuvant** (*n*=18)	***p*-value**
Focality - No. (%)				
Unifocal tumour	33 (75)	19 (73)	14 (78)	0.714
Multifocal tumour	10 (23)	7 (27)	3 (17)	
Unknown	1 (2)	0 (0)	1 (6)	
cT stage - No. (%)*				
1	20 (46)	17 (65)	3 (17)	**0.003**
2	17 (39)	7 (27)	10 (56)	
3	4 (9)	2 (8)	2 (11)	
4	2 (5)	0 (0)	2 (11)	
Unknown	1 (2)	0 (0)	1 (6)	
cG grade - No. (%)*				
1	9 (21)	7 (27)	2 (11)	0.638
2	22 (50)	10 (39)	12 (67)	
3	9 (21)	6 (23)	3 (17)	
Unknown	4 (9)	3 (12)	1 (6)	
cN stage - No. (%)*				
0	34 (77)	23 (89)	11 (61)	**0.016**
1	7 (16)	3 (12)	4 (22)	
2	1 (2)	0 (0)	1 (6)	
3	2 (5)	0 (0)	2 (11)	
cT size - in mm				
Median (IQR)	22 (16)	19 (12)	28 (16)	**0.012**
(y)pT size -				
Median (IQR)	20 (11)	21 (14)	15 (13)	0.257
25%-75%	15-26	15-29	13-25	
MIB1%				
Median (IQR)	15 (25)	20 (20)	10 (24)	0.492
25%-75%	5-30	10-30	5-29	
Ki-67%				
Mean (SD)	26 (20)	27 (17)	25 (25)	0.850
Range	2-75	5-60	2-75	
OK-type - No (%)				
Lumpectomy	20 (46)	12 (46)	8 (44)	0.954
Mastectomy	23 (52)	14 (54)	9 (50)	

Unknown	1 (2)	0 (0)	1 (6)	
ER- No (%)				
Negative	0 (0)	0 (0)	0 (0)	-
Positive	44 (100)	26 (100)	18 (100)	
ER %				
Median (IQR)	100 (0)	100 (0)	100 (5)	0.263
25%-75%	100-100	100-100	95-100	
PR- No (%)				
Negative	18 (41)	9 (35)	9 (50)	0.307
Positive	26 (59)	17 (65)	9 (50)	
PR %				
Median (IQR)	25 (79)	30 (75)	12 (91)	0.745
25%-75%	1-80	5-80	0-92	
DM-type II - No. (%)*				
No	39 (89)	23 (89)	16 (89)	1.000
Yes	5 (11)	3 (12)	2 (11)	
Prior systemic treatment - No. (%)				
No	44 (100)	26 (100)	18 (100)	-
Yes	0 (0)	0 (0)	0 (0)	
Days therapeutic antibiotic use last year				
Median (IQR)	7 (4)	6 (3)	9 (-)	0.286
25-75%	5-9	5-8	7- -	
Days from operation				
Mean (SD)	50 (23)	50 (23)	-	-
Range	18-93	18-93		
Prophylactic antibiotic use during operation - No. (%)				
No	14 (54)	14 (54)	18 (100)	**<0.001**
Yes	12 (46)	12 (46)	0 (0)	
Oral contraception use - years				
Median (IQR)	12 (13)	10 (15)	15 (19)	0.104
25%-75%	8-21	5-20	10-29	
Years between T0 faecal sample and last oral contraception use				
Mean (SD)	20 (13)	22 (14)	17 (12)	0.274
Range	0.1-49.3	0.2-49	0.1-39	
Years between T0 faecal sample and last hormone IUD use				
Mean (SD)	7 (5)	5 (5)	9 (5)	0.229
Range	1-15	1-12	5-15	

*Percentages do not add up to 100% due to rounding.

Supplementary Table 2: Longitudinal clinical characteristics of the total study population

Clinical characteristics	T0 before AC-D	T1 during AC	T2 during D	T3 after AC-D	*p*-value	Pairwise comparison
Karnofsky Performance Score - No (%)*					**<0.001**	**T0 vs T1 < 0.001**<br>**T0 vs T2 < 0.001**<br>**T0 vs T3 < 0.001**<br>T1 vs T2 = 0.051<br>T1 vs T3 = 0.531<br>**T2 vs T3 = 0.005**
20-30	0 (0)	0 (0)	1 (3)	0 (0)		
40-50	0 (0)	0 (0)	2 (6)	1 (3)		
60-70	3 (7)	9 (22)	13 (40)	11 (29)		
80-90	22 (50)	31 (74)	11 (41)	23 (61)		
100	19 (43)	2 (5)	3 (9)	3 (8)		
MUST-score - No (%)					**0.023**	T0 vs T1 = 0.117<br>T0 vs T2 = 0.022<br>T0 vs T3 = 0.739<br>**T1 vs T2 < 0.001**<br>T1 vs T3 = 0.531<br>**T2 vs T3 = 0.005**
Low risk	38 (86)	34 (79)	22 (67)	32 (84)		
Medium risk	6 (14)	5 (12)	6 (18)	6 (16)		
High risk	0 (0)	4 (9)	5 (15)	0 (0)		
BMI - kg/m^2					0.338	T0 vs T1 = 0.024<br>T0 vs T2 = 0.265<br>T0 vs T3 = 0.777<br>T1 vs T2 = 0.470<br>T1 vs T3 = 0.330<br>T2 vs T3 = 0.048
Median (IQR)	26 (5)	26 (5)	27 (5)	26 (4)		
25%-75%	24-29	23-28	24-28	24-28		

*Percentages do not add up to 100% due to rounding.
MUST: Malnutrition Universal Screening Tool.
Wilcoxon signed-rank sum test with Bonferroni correction was used for pairwise comparison. After Bonferroni correction *p*-values below 0.0125 indicated significance.

Supplementary Table 3: Longitudinal clinical data - therapeutic and prophylactic antibiotic use during the course of AC-D treatment of the total study population including the comparison between adjuvant and neoadjuvant treated patients.

**Antibiotic use**	**Total**	**Adjuvant**	**Neoadjuvant**	***p*-value**
Between T0-T1				
Antibiotic use - No (%)				0.060
No	33 (79)	17 (68)	16 (94)	
Yes	9 (21)	8 (32)	1 (6)	
Between T1-T2				
Antibiotic use - No (%)				1.000
No	18 (62)	10 (63)	8 (62)	
Yes	11 (38)	6 (38)	5 (39)	
Between T2-T3				
Antibiotic use - No (%)				1.000
No	35 (95)	20 (95)	15 (94)	
Yes	2 (5)	1 (5)	1 (6)	

Supplementary Table 4: Therapy adjustments of the total study population during the course of AC-D

**Therapy adjustments**	**T1** during AC	**T2** during D	**T3** overall
Therapy adjustments overall - No (%)			
No	37 (86)	18 (42)	14 (33)
Yes	6 (14)	25 (58)	29 (67)
Type therapy adjustments overall - No (%)			
Stop	0 (0)	16 (37)	12 (28)
Reduction, delay, and/or switch	6 (14)	9 (21)	17 (39)
% dose received overall			
Median (IQR)	100 (0)	88 (25)	94 (13)
25%-75%	100-100	75-100	88-100

N=43 since one patient did not start with AC-D.
During AC, only one patient received 95% of the planned dosses.

Supplementary Table 5: Longitudinal α-diversity measures of participants who provided all four samples (*n*=28)

**α−diversity measures**	**T0** before AC-D	**T1** during AC	**T2** during D	**T3** after AC-D	**ANOVA *p*-value**
Observed richness					
Mean (SD)	240 (48)	229 (40)	218 (57)	217 (47)	*p*=0.042
Range	136-330	157-308	84-310	87-304	
Shannon					
Mean (SD)	4.0 (0.3)	3.9 (0.3)	3.9 (0.3)	3.9 (0.3)	*p*=0.206
Range	3.3-4.6	3.4-4.5	3.1-4.4	3.1-4.5	

Changes in α-diversity measures of the 28 participants who provided all four samples before AC-D, during AC, during D, and after AC-D treatment, measured in terms of observed species richness (p=0.042; n=28) and Shannon index (p=0.206; n=28). Repeated measure ANOVA was performed to test differences in α-diversity measures over time.

**Supplementary Table 6:** Longitudinal α-diversity measures of all samples of the total population					
**α−diversity measures**	**T0**<br>before AC-D<br>(n=44)	**T1**<br>during AC<br>(n=43)	**T2**<br>during D<br>(n=29)	**T3**<br>after AC-D<br>(n=37)	**Pairwise comparison**
Observed richness<br>*Median (IQR)*<br>*25%-75%*	 247 (61)<br>203-264	 230 (75)<br>181-256	 232 (78)<br>178-255	 221 (64)<br>185-249	T0 vs T1 = 0.038<br>T0 vs T2 = 0.029<br>**T0 vs T3 = 0.003**<br>T1 vs T2 = 0.284<br>T1 vs T3 = 0.088<br>T2 vs T3 = 0.657
Shannon index<br>*Median (IQR)*<br>*25%-75%*	 4.03 (0.4)<br>3.8-4.2	 3.92 (0.5)<br>3.7-4.2	 3.90 (0.5)<br>3.7-4.2	 3.99 (0.5)<br>3.7-4.1	T0 vs T1 = 0.137<br>T0 vs T2 = 0.090<br>T0 vs T3 = 0.099<br>T1 vs T2 = 0.611<br>T1 vs T3 = 0.429<br>T2 vs T3 = 0.569

Wilcoxon signed-rank sum test with Bonferroni correction was used for pairwise comparison. After Bonferroni correction *p*-values below 0.0125 indicated significance.

**Supplementary Table 7A:** α-diversity measures in patients with or without therapeutic antibiotics 1 year prior to T0				
**α-diversity measures**	**Total** (*n*=44)	**- Antibiotics** (*n*=32)	**+ Antibiotics** (*n*=12)	***p*-value**
T0 Observed richness				
Mean (SD)	234 (45)	230 (48)	244 (39)	0.388
Range	135-330	135-330	166-301	
T1 Observed richness				
Mean (SD)	222 (47)	221 (46)	225 (52)	0.802
Range	107-308	121-293	107-308	
T2 Observed richness				
Mean (SD)	217 (57)	216 (48)	222 (78)	0.789
Range	84-310	134-294	84-310	
T3 Observed richness				
Mean (SD)	213 (48)	215 (41)	206 (66)	0.606
Range	87-304	104-280	87-304	
T0 Shannon index				
Mean (SD)	4.0 (0.3)	4.0 (0.36)	4.0 (0.29)	0.856
Range	3.0-4.6	3.0-4.6	3.6-4.5	
T1 Shannon index				
Mean (SD)	3.9 (0.4)	3.9 (0.35)	3.9 (0.43)	0.996
Range	2.8-4.5	2.8-4.5	2.8-4.5	
T2 Shannon index				
Mean (SD)	3.9 (0.3)	3.9 (0.27)	4.0 (0.45)	0.413
Range	3.1-4.4	3.3-4.4	3.1-4.4	
T3 Shannon index				
Median (IQR)	4.0 (0.5)	4.0 (0.36)	4.0 (0.66)	0.671
25-75%	3.7-4.1	3.8-4.1	3.5-4.1	

Supplementary Table 7B: alpha diversity measures with or without prophylactic antibiotics during OK all of the adjuvant treated patients

**α-diversity measures**	**Total** (*n*=26)	**- Antibiotics** (*n*=14)	**+ Antibiotics** (*n*=12)	***p*-value**
T0 Observed richness				
Median (IQR)	248 (62)	239 (61)	249 (71)	0.667
25-75%	205-267	204-264	186-258	
T0 Shannon index				
Mean (SD)	4.0 (0.25)	4.0 (0.29)	3.8 (0.29)	0.155
Range	3.5-4.5	3.9-4.2	3.7-4.0	

Supplementary Table 8: correlation between antibiotic administration during AC-D treatment and α-diversity

**α-diversity measures**	**antibiotics during T0-T1** (*n*=9, 21%)		**antibiotics during T1-T2** (*n*=11, 38%)		**antibiotics during T2-T3** (*n*=2, 5%)	
	Correlation coefficient	*p*-value	Correlation coefficient	*p*-value	Correlation coefficient	*p*-value
T1 Observed species richness	-0.457	**0.002**	NA	NA	NA	NA
T2 Observed species richness	-0.296	0.126	-0.221	0.250	NA	NA
T3 Observed species richness	-0.091	0.597	-0.092	0.640	-0.078	0.645
T1 Shannon index	-0.452	**0.003**	NA	NA	NA	NA
T2 Shannon index	-0.312	0.105	-0.195	0.310	NA	NA
T3 Shannon index	-0.233	0.171	-0.018	0.926	<0.001	1.000

Spearman's rho (r_s) correlation coefficient was used to assess the relation between antibiotic administration and α-diversity measures
NA: not applicable

**Supplementary Table 9:** correlation between cumulative antibiotic administration and α-diversity						
**α-diversity measures**	**Cumulative antibiotics until T1** (*n*=17, 40%)		**Cumulative antibiotics until T2** (*n*=17, 59%)		**Cumulative antibiotics until T3** (*n*=21, 57%)	
	Correlation coefficient	*p*-value	Correlation coefficient	*p*-value	Correlation coefficient	*p*-value
T1 Observed species richness	-0.178	0.253	NA	NA	NA	NA
T2 Observed species richness	-0.084	0.666	-0.088	0.650	NA	NA
T3 Observed species richness	-0.026	0.878	-0.089	0.651	-0.158	0.349
T1 Shannon index	-0.241	0.119	NA	NA	NA	NA
T2 Shannon index	-0.025	0.897	-0.075	0.698	NA	NA
T3 Shannon index	-0.198	0.239	-0.080	0.684	-0.225	0.181

Spearman's rho (r_s) correlation coefficient was used to assess the relation between cumulative antibiotic administration and α-diversity measures.
NA: not applicable
Cumulative antibiotic use was defined as: cumulative therapeutic and prophylactic antibiotic use from the year prior to baseline faecal sample collection until the index sample

**Supplementary Table 10:** Longitudinal differential abundant taxa of the total study population						
**Taxa**	**T0** before AC-D (*n*=44)	**T1** during AC (*n*=43)	**T2** during D (*n*=29)	**T3** after AC-D (*n*=37)	***p*-value**	**Pairwise comparison**
Proteobacteria<br>*Median (IQR)*<br>*25%-75%*	2.12 (0.94)<br>1.70-2.63	2.54 (0.69)<br>2.18-2.87	2.60 (0.67)<br>2.36-3.03	2.36 (0.98)<br>1.84-2.82	**0.006**	T0 vs T1 = 0.023<br>**T0 vs T2 < 0.001**<br>T0 vs T3 = 0.074<br>T1 vs T2 = 0.043<br>T1 vs T3 = 0.667<br>**T2 vs T3 = 0.003**
unclassified Enterobacterales<br>*Median (IQR)*<br>*25%-75%*	1.15 (1.91)<br>0.00-1.91	1.36 (2.28)<br>0.00-2.28	2.24 (1.24)<br>1.49-2.74	0.85 (1.95)<br>0.00-1.95	**<0.001**	T0 vs T1 = 0.122<br>**T0 vs T2 < 0.001**<br>T0 vs T3 = 0.877<br>**T1 vs T2 < 0.001**<br>T1 vs T3 = 0.117<br>**T2 vs T3 < 0.001**
Lactobacillus<br>*Median (IQR)*<br>*25%-75%*	0.00 (1.39)<br>0.00-1.39	0.95 (2.10)<br>0.00-2.10	1.72 (2.57)<br>0.00-2.57	1.40 (2.04)<br>0.00-2.04	**0.004**	**T0 vs T1 = 0.006**<br>**T0 vs T2 = 0.002**<br>**T0 vs T3 = 0.001**<br>T1 vs T2 = 0.024<br>T1 vs T3 = 0.245<br>T2 vs T3 = 0.174
Ruminococcaceae NK4A214 group<br>*Median (IQR)*<br>*25%-75%*	2.48 (1.25)<br>1.72-2.97	2.41 (0.97)<br>1.79-2.76	1.88 (1.91)<br>0.50-2.41	1.85 (1.36)<br>1.30-2.66	**<0.001**	T0 vs T1 = 0.132<br>**T0 vs T2 = 0.001**<br>**T0 vs T3 < 0.001**<br>**T1 vs T2 = 0.005**<br>**T1 vs T3 = 0.011**<br>T2 vs T3 = 0.927
Intestinibacter<br>*Median (IQR)*<br>*25%-75%*	2.00 (1.44)<br>1.21-2.65	1.54 (2.47)<br>0.00-2.47	2.23 (1.90)<br>1.17-3.07	2.46 (1.26)<br>1.65-2.90	0.347	T0 vs T1 = 0.013<br>T0 vs T2 = 0.200<br>T0 vs T3 = 0.219<br>T1 vs T2 = 0.041<br>**T1 vs T3 = 0.002**<br>T2 vs T3 = 0.716
Marvinbryantia<br>*Median (IQR)*<br>*25%-75%*	1.92 (0.56)<br>1.59-2.15	1.83 (0.91)<br>1.11-2.02	1.41 (1.95)<br>0.00-1.95	1.76 (1.91)<br>0.00-1.91	**0.020**	**T0 vs T1 = 0.003**<br>**T0 vs T2 < 0.001**<br>**T0 vs T3 = 0.003**<br>T1 vs T2 = 0.041<br>T1 vs T3 = 0.140<br>T2 vs T3 = 0.685
Christensenellaceae R7 group<br>*Median (IQR)*<br>*25%-75%*	2.83 (0.60)<br>2.57-3.17	2.72 (0.74)<br>2.17-2.91	2.48 (1.93)<br>0.86-2.79	2.61 (1.24)<br>1.71-2.95	**0.008**	T0 vs T1 = 0.013<br>**T0 vs T2 = 0.001**<br>**T0 vs T3 = 0.004**<br>T1 vs T2 = 0.387<br>T1 vs T3 = 0.489<br>T2 vs T3 = 0.548
Ruminococcaceae UCG-005<br>*Median (IQR)*	2.41 (0.73)	2.30 (0.73)	2.00 (1.31)	2.12 (0.65)	**<0.001**	**T0 vs T1 = 0.007**<br>**T0 vs T2 < 0.001**<br>**T0 vs T3 < 0.001**

25%-75%	2.09-2.81	1.87-2.60	1.16-2.47	1.90-2.54		**T1 vs T2 = 0.012**<br>T1 vs T3 = 0.105<br>T2 vs T3 = 0.317
Turicibacter<br>*Median (IQR)*<br>*25%-75%*	 1.42 (2.27)<br>0.00-2.27	 0.95 (1.79)<br>0.00-1.79	 1.89 (1.68)<br>0.85-2.52	 1.45 (2.26)<br>0.00-2.26	0.069	T0 vs T1 = 0.098<br>T0 vs T2 = 0.062<br>T0 vs T3 = 0.962<br>T1 vs T2 = 0.015<br>T1 vs T3 = 0.066<br>T2 vs T3 = 0.067

Friedman's ANOVA was used to test for changes in abundance over time.
Wilcoxon signed-rank sum test with Bonferroni correction was used for pairwise comparison. After Bonferroni correction *p*-values below 0.0125 indicated significance.

Supplementary Table 11: Longitudinal CTCAE in grade of the total study population

**Toxicity grade**	**T0**<br>before AC-D	**T1**<br>during AC	**T2**<br>during D	**T3**<br>after AC-D	***p*-value**	**Pairwise comparison**
Nausea - No. (%)*<br>*0*<br>*1*<br>*2*<br>*3*	 43 (98)<br>1 (2)<br>0 (0)<br>0 (0)	 23 (54)<br>15 (35)<br>4 (9)<br>1 (2)	 19 (61)<br>6 (19)<br>6 (19)<br>0 (0)	 35 (92)<br>1 (3)<br>1 (3)<br>1 (3)	**<0.001**	**T0 vs T1 < 0.001**<br>**T0 vs T2 = 0.002**<br>T0 vs T3 = 0.197<br>T1 vs T2 = 1.000<br>**T1 vs T3 = 0.005**<br>**T2 vs T3 = 0.007**
Vomiting - No (%)<br>*0*<br>*1*	 44 (100)<br>0 (0)	 42 (98)<br>1 (2)	 30 (97)<br>1 (3)	 37 (97)<br>1 (3)	0.392	T0 vs T1 = 0.317<br>T0 vs T2 = 0.317<br>T0 vs T3 = 0.317<br>T1 vs T2 = 1.000<br>T1 vs T3 = 1.000<br>T2 vs T3 = 1.000
Oral mucositis - No (%)*<br>*0*<br>*1*<br>*2*<br>*3*<br>*4*	 43 (98)<br>1 (2)<br>0 (0)<br>0 (0)<br>0 (0)	 19 (44)<br>18 (42)<br>6 (14)<br>0 (0)<br>(0)	 15 (48)<br>9 (29)<br>6 (19)<br>1 (3)<br>0 (0)	 31 (82)<br>5 (13)<br>0 (0)<br>1 (3)<br>1 (3)	**<0.001**	**T0 vs T1 < 0.001**<br>**T0 vs T2 < 0.001**<br>T0 vs T3 = 0.024<br>T1 vs T2 = 0.227<br>**T1 vs T3 = 0.009**<br>**T2 vs T3 < 0.001**
Diarrhoea - No (%)*<br>*0*<br>*1*<br>*2*<br>*3*	 44 (100)<br>0 (0)<br>0 (0)<br>0 (0)	 37 (86)<br>4 (9)<br>2 (5)<br>0 (0)	 24 (77)<br>6 (19)<br>1 (3)<br>0 (0)	 36 (95)<br>0 (0)<br>1 (3)<br>1 (3)	**0.005**	T0 vs T1 = 0.023<br>**T0 vs T2 = 0.011**<br>T0 vs T3 = 0.180<br>T1 vs T2 = 0.527<br>T1 vs T3 = 0.666<br>T2 vs T3 = 0.014
Constipation - No. (%)*<br>*0*<br>*1*<br>*2*	 42 (96)<br>2 (5)<br>0 (0)	 25 (58)<br>16 (37)<br>2 (5)	 20 (65)<br>10 (32)<br>1 (3)	 34 (90)<br>4 (11)<br>0 (0)	**<0.001**	**T0 vs T1 < 0.001**<br>**T0 vs T2 = 0.004**<br>T0 vs T3 = 0.157<br>T1 vs T2 = 0.197<br>**T1 vs T3 = 0.002**<br>**T2 vs T3 = 0.011**
Fever - No (%)*<br>*0*<br>*1*<br>*2*<br>*3*<br>*4*	 44 (100)<br>0 (0)<br>0 (0)<br>0 (0)<br>0 (0)	 40 (93)<br>2 (5)<br>0 (0)<br>0 (0)<br>1 (2)	 27 (87)<br>2 (7)<br>2 (7)<br>0 (0)<br>0 (0)	 36 (95)<br>2 (5)<br>0 (0)<br>0 (0)<br>0 (0)	0.072	T0 vs T1 = 0.102<br>T0 vs T2 = 0.063<br>T0 vs T3 = 0.157<br>T1 vs T2 = 1.000<br>T1 vs T3 = 0.414<br>T2 vs T3 = 0.102
Peripheral sensory neuropathy - No (%)<br>*0*<br>*1*<br>*2*<br>*3*	 43 (98)<br>0 (0)<br>1 (2)<br>0 (0)	 34 (79)<br>8 (19)<br>1 (2)<br>0 (0)	 3 (10)<br>19 (61)<br>8 (26)<br>1 (3)	 7 (18)<br>22 (58)<br>8 (21)<br>1 (3)	**<0.001**	T0 vs T1 = 0.021<br>**T0 vs T2 < 0.001**<br>**T0 vs T3 < 0.001**<br>**T1 vs T2 < 0.001**<br>**T1 vs T3 < 0.001**<br>T2 vs T3 = 0.132
Hand foot syndrome - No. (%)*<br>*0*	 43 (98)	 35 (81)	 15 (48)	 20 (54)	**<0.001**	T0 vs T1 = 0.083<br>**T0 vs T2 = 0.002**

1<br>*2*<br>*3*	0 (0)<br>1 (2)<br>0 (0)	8 (19)<br>0 (0)<br>0 (0)	11 (36)<br>4 (13)<br>1 (3)	12 (32)<br>3 (8)<br>2 (5)		**T0 vs T3 < 0.001**<br>**T1 vs T2 = 0.002**<br>**T1 vs T3 = 0.006**<br>T2 vs T3 = 0.323
Fatigue - No (%)*<br>*0*<br>*1*<br>*2*<br>*3*	 29 (66)<br>15 (34)<br>0 (0)<br>0 (0)	 2 (5)<br>31 (72)<br>10 (23)<br>0 (0)	 0 (0)<br>18 (58)<br>11 (36)<br>2 (7)	 3 (8)<br>27 (71)<br>8 (21)<br>0 (0)	**<0.001**	**T0 vs T1 < 0.001**<br>**T0 vs T2 < 0.001**<br>**T0 vs T3 < 0.001**<br>**T1 vs T2 = 0.008**<br>T1 vs T3 = 0.593<br>**T2 vs T3 = 0.005**
Alopecia - No (%)*<br>*0*<br>*1*<br>*2*	 44 (100)<br>0 (0)<br>0 (0)	 2 (5)<br>8 (19)<br>33 (77)	 4 (13)<br>6 (19)<br>21 (68)	 13 (34)<br>2 (5)<br>23 (61)	**<0.001**	**T0 vs T1 < 0.001**<br>**T0 vs T2 < 0.001**<br>**T0 vs T3 < 0.001**<br>T1 vs T2 = 0.163<br>**T1 vs T3 = 0.007**<br>**T2 vs T3 = 0.006**

*Percentages do not add up to 100% due to rounding.
Friedman's ANOVA was used to indicate differences in chemotherapy toxicity during the course of AC-D
Wilcoxon signed-rank sum test with Bonferroni correction was used for pairwise comparison. After Bonferroni correction *p*-values below 0.0125 indicated significance.

Supplementary Table 12: Longitudinal clinical characteristics of the total study population (*N*=44) - bone marrow toxicity

**Bone marrow toxicity**	**T0**<br>before AC-D<br>(*n*=44)	**T1**<br>during AC<br>(*n*=43)	**T2**<br>during D<br>(*n*=29)	**T3**<br>after AC-D<br>(*n*=37)	***p*-value**	**Pairwise comparison**
Haemoglobin - in µ/L<br>*Median (IQR)*<br>*25%-75%*	 8.4 (0.7)<br>8.1-8.8	 7.3 (0.9)<br>6.7-7.6	 7.1 (0.7)<br>6.7-7.4	 6.9 (0.7)<br>6.7-7.4	**<0.001**	**T0 vs T1 < 0.001**<br>**T0 vs T2 < 0.001**<br>**T0 vs T3 < 0.001**<br>T1 vs T2 = 0.030<br>T1 vs T3 = 0.464<br>T2 vs T3 = 0.374
Thrombocytes - in 10^9/l<br>*Median (IQR)*<br>*25%-75%*	 272 (92)<br>255-347	 323 (158)<br>250-405	 331 (108)<br>259-367	 317 (132)<br>260-392	**0.012**	T0 vs T1 = 0.111<br>T0 vs T2 = 0.280<br>T0 vs T3 = 0.465<br>T1 vs T2 = 0.308<br>T1 vs T3 = 0.082<br>T2 vs T3 = 0.030
Leucocytes - in 10^9/l<br>*Median (IQR)*<br>*25%-75%*	 7.2 (3.1)<br>5.9-9.0	 6.9 (7.4)<br>4.6-12	 7.9 (6.2)<br>5.3-11.5	 6.8 (3.6)<br>5.2-8.8	0.151	T0 vs T1 = 0.621<br>T0 vs T2 = 0.256<br>T0 vs T3 = 0.455<br>T1 vs T2 = 0.993<br>T1 vs T3 = 0.092<br>T2 vs T3 = 0.041
Neutrophils - 10^9/l<br>*Median (IQR)*<br>*25%-75%*	 4.6 (2.5)<br>3.3-5.8	 4.6 (7.4)<br>2.9-10.3	 6.0 (6.6)<br>3.5-10.1	 4.3 (2.0)<br>3.4-5.4	0.190	T0 vs T1 = 0.350<br>T0 vs T2 = 0.104<br>T0 vs T3 = 0.600<br>T1 vs T2 = 0.801<br>T1 vs T3 = 0.056<br>T2 vs T3 = 0.016

Friedman's ANOVA was used to indicate differences in bone marrow toxicity during the course of AC-D
Wilcoxon signed-rank sum test with Bonferroni correction was used for pairwise comparison. After Bonferroni correction *p*-values below 0.0125 indicated significance.

**Supplementary Table 13:** β-diversity and chemotherapy toxicity of the total study population				
	**T1** during AC (*n*=43)		**T2** during D (*n*=29)	
**Any grade CTCAE**	**Genus** *p*-value	**Phylum** *p*-value	**Genus** *p*-value	**Phylum** *p*-value
Diarrhoea	0.4656	0.6536	0.1584	0.3597
Nausea	0.9138	0.6290	0.6312	0.2483
Oral mucositis	0.4268	0.6718	0.8789	0.4593
Hand foot syndrome	0.8237	0.9645	0.8926	0.3600
Peripheral sensory neuropathy	0.8630	0.9962	0.6169	0.4959

PERMANOVA showed that microbial community structures on both phylum and genus level during AC and during D were not associated with toxicity.

**Supplementary Table 14:** Longitudinal clinical characteristics of neoadjuvant treated patients						
**Clinical characteristics**	**T0** before AC-D	**T1** during AC	**T2** during D	**T3** after AC-D	***p*-value**	**Pairwise comparison**
Karnofsky Performance Score - No (%)*					**<0.001**	**T0 vs T1 < 0.001**<br>**T0 vs T2 = 0.001**<br>**T0 vs T3 = 0.001**<br>T1 vs T2 = 0.031<br>T1 vs T3 = 0.480<br>**T2 vs T3 = 0.013**
40-50	0 (0)	0 (0)	1 (7)	0 (0)		
60-70	0 (0)	3 (18)	6 (43)	4 (25)		
80-90	5 (28)	13 (77)	6 (43)	12 (76)		
100	13 (72)	1 (6)	1 (7)	0 (0)		
MUST-score - No (%)*					0.294	T0 vs T1 = 0.157<br>T0 vs T2 = 0.129<br>T0 vs T3 = 0.180<br>T1 vs T2 = 0.450<br>T1 vs T3 = 1.000<br>T2 vs T3 = 0.083
Low risk	16 (89)	14 (78)	11 (73)	12 (75)		
Medium risk	2 (11)	2 (11)	2 (13)	4 (25)		
High risk	0 (0)	2 (11)	2 (13)	0 (0)		
BMI - kg/m^2					0.332	T0 vs T1 = 0.494<br>T0 vs T2 = 0.865<br>T0 vs T3 = 0.755<br>T1 vs T2 = 0.753<br>T1 vs T3 = 0.348<br>T2 vs T3 = 0.154
Median (IQR)	26 (7)	27 (7)	27 (6)	27 (5)		
25%-75%	24-31	23-30	24-29	24-29		

* Percentages do not add up to 100% due to rounding
Wilcoxon signed-rank sum test with Bonferroni correction was used for pairwise comparison. After Bonferroni correction *p*-values below 0.0125 indicated significance.

Supplementary Table 15: Longitudinal CTCAE in grade of neo-adjuvant treated patients

**Toxicity grade**	**T0**<br>before AC-D	**T1**<br>during AC	**T2**<br>during D	**T3**<br>after AC-D	***p*-value**	**Pairwise comparison**
Nausea - No. (%)					**0.001**	T0 vs T1 = 0.025<br>T0 vs T2 = 0.014<br>T0 vs T3 = 1.000<br>T1 vs T2 = 0.096<br>T1 vs T3 = 0.046<br>T2 vs T3 = 0.014
0	18 (100)	13 (72)	7 (50)	16 (100)		
1	0 (0)	5 (28)	5 (36)	0 (0)		
2	0 (0)	0 (0)	2 (14)	0 (0)		
3	0 (0)	0 (0)	0 (0)	0 (0)		
Vomiting - No (%)					-	-
0	18 (100)	18 (100)	14 (100)	16 (100)		
1	0 (0)	0 (0)	0 (0)	0 (0)		
Oral mucositis - No (%)*					**0.002**	**T0 vs T1 = 0.003**<br>T0 vs T2 = 0.015<br>T0 vs T3 = 0.157<br>T1 vs T2 = 0.603<br>T1 vs T3 = 0.035<br>T2 vs T3 = 0.014
0	18 (100)	8 (44)	7 (50)	14 (88)		
1	0 (0)	8 (44)	4 (29)	2 (13)		
2	0 (0)	2 (11)	3 (21)	0 (0)		
3	0 (0)	0 (0)	0 (0)	0 (0)		
4	0 (0)	0 (0)	0 (0)	0 (0)		
Diarrhoea - No (%)*					**0.019**	T0 vs T1 = 0.180<br>T0 vs T2 = 0.046<br>T0 vs T3 = 1.000<br>T1 vs T2 = 0.083<br>T1 vs T3 = 0.317<br>T2 vs T3 = 0.046
0	18 (100)	16 (89)	10 (71)	16 (100)		
1	0 (0)	1 (6)	4 (29)	0 (0)		
2	0 (0)	1 (6)	0 (0)	0 (0)		
3	0 (0)	0 (0)	0 (0)	0 (0)		
Constipation - No. (%)					**0.019**	**T0 vs T1 = 0.008**<br>T0 vs T2 = 0.046<br>T0 vs T3 = 1.000<br>T1 vs T2 = 0.705<br>T1 vs T3 = 0.025<br>T2 vs T3 = 0.046
0	18 (100)	11 (61)	10 (71)	16 (100)		
1	0 (0)	7 (39)	4 (29)	0 (0)		
2	0 (0)	0 (0)	0 (0)	0 (0)		
Fever - No (%)*					0.194	T0 vs T1 = 0.180<br>T0 vs T2 = 0.157<br>T0 vs T3 = 1.000<br>T1 vs T2 = 0.785<br>T1 vs T3 = 0.180<br>T2 vs T3 = 0.157
0	18 (100)	16 (89)	12 (86)	16 (100)		
1	0 (0)	1 (6)	0 (0)	0 (0)		
2	0 (0)	0 (0)	2 (14)	0 (0)		
3	0 (0)	0 (0)	0 (0)	0 (0)		
4	0 (0)	1 (6)	0 (0)	0 (0)		
Peripheral sensory neuropathy - No (%)*					**<0.001**	T0 vs T1 = 0.102<br>**T0 vs T2 = 0.001**<br>**T0 vs T3 = 0.001**<br>T1 vs T2 = 0.013<br>**T1 vs T3 = 0.009**<br>T2 vs T3 = 0.655
0	18 (100)	15 (83)	2 (14)	3 (19)		
1	0 (0)	2 (11)	9 (64)	10 (63)		
2	0 (0)	1 (6)	3 (21)	3 (19)		
3	0 (0)	0 (0)	0 (0)	0 (0)		
Hand foot syndrome - No. (%)*					**0.014**	T0 vs T1 = 0.046<br>T0 vs T2 = 0.024<br>T0 vs T3 = 0.039<br>T1 vs T2 = 0.063<br>T1 vs T3 = 0.336
0	18 (100)	14 (78)	8 (57)	11 (69)		
1	0 (0)	4 (22)	3 (21)	3 (19)		
2	0 (0)	0 (0)	3 (21)	1 (6)		

3	0 (0)	0 (0)	0 (0)	1 (6)		T2 vs T3 = 0.157
Fatigue - No (%)*<br>*0*<br>*1*<br>*2*<br>*3*	 14 (78)<br>4 (22)<br>0 (0)<br>0 (0)	 0 (0)<br>12 (67)<br>6 (33)<br>0 (0)	 0 (0)<br>8 (57)<br>6 (43)<br>0 (0)	 2 (13)<br>11 (69)<br>3 (19)<br>0 (0)	**<0.001**	**T0 vs T1 < 0.001**<br>**T0 vs T2 = 0.001**<br>**T0 vs T3 = 0.002**<br>T1 vs T2 = 0.317<br>T1 vs T3 = 0.102<br>T2 vs T3 = 0.034
Alopecia - No (%)*<br>*0*<br>*1*<br>*2*	 18 (100)<br>0 (0)<br>0 (0)	 2 (11)<br>5 (28)<br>11 (61)	 0 (0)<br>5 (36)<br>9 (64)	 7 (44)<br>2 (13)<br>7 (44)	**<0.001**	**T0 vs T1 < 0.001**<br>**T0 vs T2 = 0.001**<br>T0 vs T3 = 0.005<br>T1 vs T2 = 0.705<br>T1 vs T3 = 0.132<br>T2 vs T3 = 0.034

* Percentages do not add up to 100% due to rounding
Wilcoxon signed-rank sum test with Bonferroni correction was used for pairwise comparison. After Bonferroni correction *p*-values below 0.0125 indicated significance.

Supplementary Table 16: Longitudinal clinical data - therapy adjustments of neoadjuvant treated patients (*n*=18)

**Therapy adjustments**	**T1** during AC	**T2** during D	**T3** overall
Therapy adjustments overall - No (%)			
No	14 (78)	11 (61)	8 (44)
Yes	4 (22)	7 (39)	10 (56)
Type therapy adjustments overall - No (%)			
Stop	0 (0)	4 (22)	4 (22)
Reduction, delay, and/or switch	4 (22)	3 (17)	6 (33)
% dose received overall			
Median (IQR)	100 (0)	100 (20)	100 (10)
25%-75%	100-100	80-100	90-100

Supplementary Table 17: Response to AC-D of the neoadjuvant treated patients

Response to AC-D	Total	N
After AC-D response tumour pathology category - No. (%)		
Complete pathologic response	1 (6)	17
EUSOMA 2 (i)	6 (35)	
EUSOMA 2 (ii)	4 (24)	
EUSOMA 2 (iii)	6 (35)	
After AC-D pathologic response*		
Low responders	10 (59)	17
High responders	7 (41)	

* High-responders (<10% remaining tumour cells) were defined as EUSOMA 1 and EUSOMA 2 (i). Low-responders (≥10 remaining tumour cells) were defined as EUSOMA 2 (ii), EUSOMA 2 (iii), and EUSOMA 3.

**Supplementary Table 18:** Clinical characteristics of the neoadjuvant study population at baseline. Response measured after AC-D according to EUSOMA				
**Baseline characteristics**	**Total** ***n*=18**	**High responders** ***n*=7**	**Low responders** ***n*=10**	***p*-value**
Age - Years				
Mean (SD)	58 (5)	60 (5)	57 (5)	0.199
Range	49-71	55-71	49-65	
BMI - kg/m^2				
Mean (SD)	28 (6)	27 (4)	29 (6)	0.429
Range	20-42	23-33	23-42	
Weight loss past 3-6 months - in kg				
<5%	16 (89)	6 (86)	10 (100)	-
5%-10%	2 (11)	1 (14)	0 (0)	
Focality - No. (%)*				
Unifocal tumour	14 (78)	6 (86)	8 (80)	1.000
Multifocal tumour	3 (17)	1 (14)	2 (20)	
Unknown	1 (6)	0 (0)	0 (0)	
cT stage - No. (%)*				
1	3 (17)	2 (29)	1 (10)	0.325
2	10 (56)	2 (29)	8 (80)	
3	2 (11)	1 (14)	1 (10)	
4	2 (11)	2 (29)	0 (0)	
Unknown	1 (6)	0 (0)	0 (0)	
cG grade - No. (%)*				
1	2 (11)	1 (14)	1 (10)	0.621
2	12 (67)	4 (57)	7 (70)	
3	3 (17)	2 (29)	1 (10)	
Unknown	1 (6)	0 (0)	1 (0)	
cN stage - No. (%)*				
0	11 (61)	4 (57)	7 (70)	0.665
1	4 (22)	2 (29)	2 (20)	
2	1 (6)	0 (0)	0 (0)	
3	2 (11)	1 (14)	1 (10)	
cT size - in mm				
Median (IQR)	28 (16)	24 (20)	29 (16)	0.812
25%-75%	22-38	20-40	22-38	
MIB1%				
Median (IQR)	10 (24)	50 (-)	10 (17)	0.190
25%-75%	5-29	25- -	4-20	
Ki-67%				
Median (IQR)	25 (25)	50 (-)	10 (24)	0.190
25%-75%	5-30	25- -	4-28	

Tumour-type - No (%)				
Invasive carcinoma of no special type (NST)	16 (89)	7 (100)	8 (80)	0.485
Lobular	2 (11)	0 (0)	2 (20)	
Mucinous	0 (0)	0 (0)	0 (0)	
Unknown	0 (0)	0 (0)	0 (0)	
ER- No (%)				
Negative	0 (0)	0 (0)	0 (0)	-
Positive	18 (100)	7 (100)	10 (100)	
ER %				
Median (IQR)	100 (5)	100 (5)	100 (6)	0.962
25%-75%	95-100	95-100	94-100	
PR- No (%)*				
Negative	9 (50)	4 (57)	4 (40)	0.637
Positive	9 (50)	3 (43)	6 (60)	
PR %				
Median (IQR)	12 (91)	8 (80)	33 (95)	0.417
25%-75%	0-92	0-80	1-96	
Radiotherapy received any time - No. (%)*				
No	3 (17)	2 (29)	1 (10)	0.537
Yes	15 (83)	5 (71)	9 (90)	
Karnofsky Performance Score - No (%)*				
80-90	5 (28)	2 (28)	3 (30)	0.935
100	13 (72)	5 (71)	7 (70)	
MUST-score - No (%)				
Low risk	16 (89)	6 (86)	10 (100)	0.232
Medium risk	2 (11)	1 (14)	0 (0)	
High risk	0 (0)	0 (0)	0 (0)	
DM-type II - No. (%)*				
No	16 (89)	6 (86)	9 (90)	1.000
Yes	2 (11)	1 (14)	1 (10)	
Prior systemic treatment - No. (%)				
No	18 (100)	7 (100)	10 (100)	-
Yes	0 (0)	0 (0)	0 (0)	
Therapeutic antibiotic use last year - No. (%)*				
No	14 (78)	6 (86)	8 (80)	1.000
Yes	4 (22)	1 (14)	2 (20)	
Days therapeutic antibiotic use last year				
Median (IQR)	9 (-)	-	9 (-)	-
25%-75%	7- -		7- -	

*Percentages do not add up to 100% due to rounding.

Supplementary Table 19: Clinical characteristics after neoadjuvant chemotherapy. Response measured after AC-D according to EUSOMA

**Clinical characteristics**	**Total** *n*=18	**High responders** *n*=7	**Low responders** *n*=10	***p*-value**
OK-type - No (%)*				
Lumpectomy	8 (44)	3 (43)	5 (50)	1.000
Mastectomy	9 (50)	4 (57)	5 (50)	
Unknown	1 (6)	0 (0)	0 (0)	
pG grade - No. (%)*				
1	4 (22)	1 (14)	3 (30)	0.251
2	5 (28)	2 (29)	3 (30)	
3	1 (6)	1 (14)	0 (0)	
Unknown	8 (44)	3 (43)	4 (40)	
pN stage - No. (%)*				
0	9 (50)	4 (57)	5 (50)	0.690
1	6 (33)	3 (43)	3 (30)	
2	2 (11)	0 (0)	1 (10)	
3	0 (0)	0 (0)	0 (0)	
Unknown	1 (6)	0 (0)	1 (10)	
pT size - in mm				
Mean (SD)	21 (14)	19 (15)	22 (13)	0.638
Range	0-48	0-47	6-48	
pT stage - No. (%)*				
0	1 (6)	1 (14)	0 (0)	0.232
1	9 (50)	4 (57)	5 (50)	
2	7 (39)	2 (29)	5 (50)	
3	0 (0)	0 (0)	0 (0)	
4	0 (0)	0 (0)	0 (0)	
Unknown	1 (6)	0 (0)	0 (0)	
T2 Tumour reduction in %				
Mean (SD)	36 (29)	39 (39)	33 (21)	0.682
Range	-20-100	-20-100	-5-73	

*Percentages do not add up to 100% due to rounding.

Supplementary Table 20: α-diversity measures of the neoadjuvant study population. Response measured after AC-D according to EUSOMA

**α-diversity measures**	**Total** *n*=18	**High responders** *n*=7	**Low responders** *n*=10	***p*-value**
T0 Observed richness				
Mean (SD)	238 (52)	232 (70)	242 (41)	0.708
Range	136-330	136-330	153-302	
T1 Observed richness				
Mean (SD)	233 (34)	232 (45)	234 (29)	0.907
Range	169-293	172-293	169-266	
T2 Observed richness				
Mean (SD)	218 (48)	211 (31)	225 (57)	0.674
Range	136-294	175-239	136-294	
T3 Observed richness				
Mean (SD)	218 (48)	212 (36)	210 (48)	0.931
Range	136-294	178-257	126-260	
T0 Shannon index				
Mean (SD)	4.1 (0.4)	3.9 (0.6)	4.2 (0.3)	0.238
Range	3.0-4.6	3.0-4.6	3.6-4.6	
T1 Shannon index				
Mean (SD)	4.0 (0.3)	4.0 (0.3)	4.1 (0.3)	0.669
Range	3.4-4.5	3.4-4.2	3.6-4.5	
T2 Shannon index				
Mean (SD)	3.9 (0.3)	3.9 (0.2)	3.9 (0.30)	0.999
Range	3.5-4.4	3.7-4.2	3.5-4.4	
T3 Shannon index				
Median (IQR)	3.9 (0.4)	3.8 (0.3)	4.1 (0.5)	0.456
25-75%	3.7-4.1	3.7-4.0	3.7-4.1	

Differences in α-diversity between high and low responders measured at T2 according to EUSOMA were analysed with an unpaired t-test.
High-responders (<10% remaining tumour cells) were defined as EUSOMA 1 and EUSOMA 2 (i). Low-responders (≥10 remaining tumour cells) were defined as EUSOMA 2 (ii), EUSOMA 2 (iii), and EUSOMA

Supplementary Table 21: Basic 16S rRNA gene sequencing statistics of the gut bacterial microbiota using the Illumina MiSeq instrument

Item	Data
Amplified region	515F-806R
Primer sequence 515F	5‘-GTGCCAGCMGCCGCGGTAA-3‘
Primer sequence 806R	5‘-GGACTACHVGGGTWTCTAAT-3‘
Number of samples	153
Total raw reads	18.890.872
Mean reads per sample	123.470
Total sequences per sample	
Minimum	51.119
Maximum	176.490
Mean %GC	53
Sequence length	251
Sequences flagged as poor quality	0
